# High-Performance Path Tracking of a 4WD Autonomous Vehicle Using NMPC with Virtual 4WD Torque Distribution

**DOI:** 10.3390/s26144442

**Published:** 2026-07-13

**Authors:** Duc Hiep Vu, Chih-Keng Chen, Jiageng Ruan

**Affiliations:** 1 Department of Vehicle Engineering, National Taipei University of Technology, Taipei 10604, Taiwan; t113569406@ntut.edu.tw; 2 College of Mechanical and Energy Engineering, Beijing University of Technology, Beijing 100020, China

**Keywords:** trajectory optimization, nonlinear model predictive control (NMPC), virtual 4WD, torque distribution

## Abstract

This study proposes a reduced-complexity nonlinear model predictive control (NMPC) framework for high-performance path tracking of a four-wheel-drive (4WD) autonomous vehicle. A 4WD sports car equipped with four independent wheel motors is used as the test vehicle. Although the vehicle has four motors, the proposed NMPC directly optimizes the front-wheel steering command and the rear-left and rear-right wheel torque commands, while the front-wheel torques are generated using a gain-based virtual 4WD distribution law. Trajectory optimization (TRO) is performed offline to generate the reference racing line and velocity profile, while the online NMPC controller tracks the optimized reference trajectory using the front-wheel steering command and the rear-left and rear-right wheel torque commands as control inputs. This structure reduces the control complexity while maintaining the ability to improve traction utilization and yaw response. Under the investigated simulation conditions on the Shanghai International Circuit, the proposed reduced-dimensional NMPC with rear-dominant virtual 4WD torque distribution reduces the simulated lap time while maintaining bounded path-tracking errors and satisfying the track-boundary constraints. As the torque distribution gain $K_{r}$ increases from 0 to 0.5, the lap time is reduced by approximately 10.3% (from 182.08 s to 163.30 s), while the maximum lateral tracking error remains below 0.33 m and the maximum heading-angle error remains below 2.95 deg for all stable cases. However, further increasing $K_{r}$ beyond 0.5 leads to degraded tracking performance or loss of stable path following because excessive front-wheel longitudinal force reduces the available lateral tire force for steering. These results indicate that an appropriate torque distribution gain can improve corner-exit acceleration and overall lap-time performance, whereas excessive front torque assistance may degrade tracking accuracy and vehicle stability.

## 1. Introduction

Autonomous driving has attracted significant attention in recent years due to its potential to improve safety, efficiency, and mobility. However, high-speed path tracking along curved trajectories remains a significant challenge, particularly near the handling limits. In such conditions, strong nonlinear tire dynamics, actuator constraints, and coupled longitudinal-lateral vehicle behavior severely degrade tracking performance. Conventional motion planning and control frameworks typically adopt a hierarchical structure, where trajectory generation and tracking control are designed separately. Although this approach simplifies implementation, it often leads to suboptimal performance in aggressive driving scenarios, as the generated trajectory may not fully account for vehicle dynamic constraints during execution. To overcome this limitation, integrated trajectory planning and control strategies have been proposed. For instance, Srinivasan et al. [1] developed an NMPC-based framework that jointly considers planning and control, demonstrating superior performance compared to professional human drivers in racing scenarios. Similarly, Li et al. [2] proposed a time-optimal trajectory planning and tracking (TOTPT) framework, combining offline trajectory optimization with online NMPC tracking to achieve high-performance autonomous driving.

In addition to NMPC-based planning and tracking, recent nonlinear and robust control studies have emphasized the importance of disturbance rejection, parameter uncertainty handling, and stability analysis for constrained nonlinear systems. Adaptive and disturbance-observer-based sliding-mode control methods have been developed to improve robustness against lumped disturbances, model uncertainties, and load-dependent perturbations [3,4]. For vehicle systems, uncertain states and tire parameters can also degrade tracking performance; therefore, state–parameter estimation methods have been investigated to estimate sideslip angle, lateral acceleration, and tire cornering stiffness under time-varying conditions [5]. More directly related to autonomous vehicle path tracking, robust predictive control based on a polytopic vehicle model has been proposed to handle time-varying longitudinal velocity, model uncertainty, and external disturbances [6]. These studies show that robustness and constraint handling are essential for reliable path tracking. However, high-speed racing near the handling limits further requires simultaneous consideration of trajectory feasibility, tire-force saturation, longitudinal–lateral coupling, actuator constraints, and traction utilization.

Among advanced control techniques, nonlinear model predictive control (NMPC) has become a widely adopted approach for high-performance vehicle control due to its ability to explicitly handle nonlinear dynamics and constraints [7]. NMPC has been successfully applied to path tracking and autonomous racing problems, where it optimizes control inputs while respecting tire–road adhesion limits and actuator bounds. However, many existing NMPC implementations focus primarily on steering control or simplified drivetrain models, without fully exploiting the capabilities of multi-motor systems. With the increasing development of electric vehicles equipped with multiple independent motors, torque vectoring has emerged as an effective technique to enhance vehicle handling and stability. By distributing driving and braking torques among individual wheels, additional yaw moments can be generated to improve cornering performance and traction utilization. Several studies have demonstrated the benefits of torque vectoring in improving vehicle dynamics [8,9]. Nevertheless, incorporating full torque distribution into NMPC significantly increases computational complexity, making real-time implementation challenging, especially for high-speed applications. Most torque-vectoring systems reported in the literature focus on generating a corrective yaw moment in order to regulate the vehicle yaw response or to track a desired yaw-rate demand. For vehicles equipped with multiple electric motors, particularly distributed-drive configurations, left/right torque vectoring can be achieved by independently controlling the wheel torques on the front and/or rear axles. Recent studies have shown that, for distributed-drive electric vehicles, drive torque distribution can significantly enhance yaw control performance and improve vehicle stability, particularly under high lateral acceleration conditions [10,11,12]. In addition to left/right torque vectoring, front/rear torque distribution has also been investigated as an effective approach to improve vehicle behavior near the limits of handling. Model predictive control (MPC)-based strategies have been proposed to coordinate longitudinal and lateral dynamics by optimally allocating driving forces between the front and rear axles. Such approaches are particularly effective in scenarios involving high-speed cornering or over-speeding, where appropriate torque allocation can reduce path deviation and enhance overall vehicle stability [13,14,15].

Existing autonomous racing and high-speed path-tracking studies can be broadly divided into three groups. The first group focuses on trajectory optimization or minimum-lap-time planning, where vehicle dynamic constraints are considered during the offline generation of the racing line and velocity profile [1,2]. However, these methods usually do not directly address the online tracking problem or the actuator-allocation structure. The second group focuses on MPC/NMPC-based path tracking, where nonlinear vehicle dynamics and actuator constraints are handled online [6,7]. Although these methods are suitable for constrained path tracking, many formulations mainly use steering control or simplified drivetrain representations. The third group focuses on torque-vectoring or torque-allocation control for distributed-drive electric vehicles [6,7,8,9,10,11]. These methods can improve yaw response and vehicle stability, but full four-wheel torque allocation may significantly increase the online optimization dimension and computational burden.

Compared with these studies, the core innovation of the present work is the reduced-dimensional NMPC formulation with rear-dominant virtual 4WD torque distribution. Instead of optimizing four independent wheel torques online, the proposed NMPC optimizes only the front-wheel steering command and the rear-left/rear-right wheel torque commands, while the front-wheel torques are generated through a proportional virtual distribution law. Therefore, the proposed method exploits the traction benefit of a 4WD vehicle without introducing four independent wheel-torque decision variables into the online NMPC problem. This structure reduces the online optimization dimension and supports real-time-oriented implementation while retaining the execution-level four-wheel-drive torque capability.

In this study, rear-dominant virtual 4WD torque distribution refers to the execution-level torque distribution architecture rather than four independent wheel-torque decision variables in the online NMPC problem. The online NMPC directly optimizes the front-wheel steering command and the rear-left/rear-right wheel torque commands, while the front-left and front-right wheel torques are generated from the corresponding rear-wheel torque commands through the proportional gain $K_{r}$. Therefore, the NMPC control degrees of freedom are the steering command and the two rear-wheel torque commands, whereas the four-wheel-drive torque capability is realized at the actuator execution layer.

The main contributions are as follows:

- A reduced-dimensional NMPC framework is proposed for high-speed path tracking of a four-wheel-drive autonomous vehicle. The controller optimizes only the front-wheel steering command and the rear-left/rear-right wheel torque commands, thereby reducing the online optimization dimension compared with a conventional full four-wheel torque NMPC formulation.
- A rear-dominant virtual 4WD torque distribution strategy is introduced. In this strategy, the front-left and front-right wheel torques are generated proportionally from the corresponding rear-wheel torque commands through an execution-level distribution law, allowing the four-wheel-drive torque capability to be realized without adding front-wheel torque decision variables to the online NMPC problem.
- An integrated TRO–NMPC framework is developed. The offline TRO generates a near-limit minimum-lap-time reference trajectory, including the racing line, velocity profile, and sideslip-angle profile, while the online NMPC tracks the TRO-generated reference under vehicle, actuator, tire-force, motor-power, and track-boundary constraints.
- The computational benefit of the reduced-dimensional formulation is evaluated by comparing the proposed NMPC with a conventional full four-wheel torque NMPC formulation in terms of decision-variable count, dynamic equality constraints, input-rate decision variables, and solver execution time.
- The effectiveness and limitations of the proposed framework are evaluated through closed-loop numerical simulations on the Shanghai International Circuit. The results show that, under the investigated simulation conditions, the proposed rear-dominant virtual 4WD strategy reduces the simulated lap time while maintaining bounded tracking errors and satisfying the track-boundary constraints.

This paper is organized as follows. Section 2 presents the reference path generation method. Section 3 describes the path-relative kinematic model of the vehicle. Section 4 presents the nonlinear vehicle dynamics model for path tracking, including the coupled longitudinal–lateral and yaw dynamics, nonlinear tire force modeling, and the representation of distributed longitudinal forces from multiple electric motors, enabling accurate characterization of vehicle behavior near the limits of handling. Section 5 presents the overall control architecture. Section 6 formulates the trajectory optimization problem for offline trajectory planning using a direct collocation method. Section 7 presents the NMPC framework with virtual 4WD torque distribution, including the prediction model, reference generation, cost function, and constraints. Section 8 presents simulation results on the Shanghai International Circuit. Finally, Section 9 concludes the paper.

## 2. Reference Path Generation

Cubic spline interpolation is applied to the original waypoints to construct a smooth reference path. The interpolation procedure was implemented in MATLAB R2024b. The original racetrack waypoints were first parameterized by arc length and then resampled on a uniform station grid using the MATLAB ‘interp1’ function with the ‘spline’ option. The interpolated path coordinates and boundary-width data were used to construct the smooth arc-length-parameterized reference path for both the TRO and NMPC formulations. This smoothness is achieved through continuity in the lower-order derivatives of the spline function. To avoid ambiguity between the global path station and the local spline variable, each spline segment is expressed using a local coordinate. For the i-th spline segment, the local coordinate is defined as $\xi_{i}=\;s-s_{i}$, with $s\in \left[s_{i},s_{i+1}\right]$, where s is the global arc-length coordinate and $s_{i}$ is the left knot of the i-th segment. The cubic spline polynomial is then defined over the local interval $\xi_{i}\in \left[0,\;s_{i+1}-s_{i}\right]$ as follows [16]:

(1) $$\begin{array}{c} f_{i}\left(\xi_{i}\right)=a_{i}\xi_{i}^{3}+b_{i}\xi_{i}^{2}+c_{i}\xi_{i}+d_{i},i=1,\ldots ,N_{seg}\\ f_{i}^{\prime }\left(\xi_{i}\right)=3a_{i}\xi_{i}^{2}+2b_{i}\xi_{i}+c_{i},i=1,\ldots ,N_{seg}\\ f_{i}^{\prime \prime }\left(\xi_{i}\right)=6a_{i}\xi_{i}+2b_{i},i=1,\ldots ,N_{seg} \end{array}$$

Here, $f_{i}^{\prime }\left(\xi_{i}\right)$ and $f_{i}^{\prime \prime }\left(\xi_{i}\right)$ denote the first and second derivatives with respect to the local coordinate $\xi_{i}$. Since $\frac{d\xi_{i}}{ds}=1$, these derivatives are equivalent to the derivatives with respect to the global arc-length coordinate within the corresponding spline segment. Using the local coordinate $\xi_{i}=\;s-s_{i}$ avoids ambiguity between the global path station and the local spline variable and reduces potential numerical-conditioning issues in the polynomial representation.

**Figure 1:**
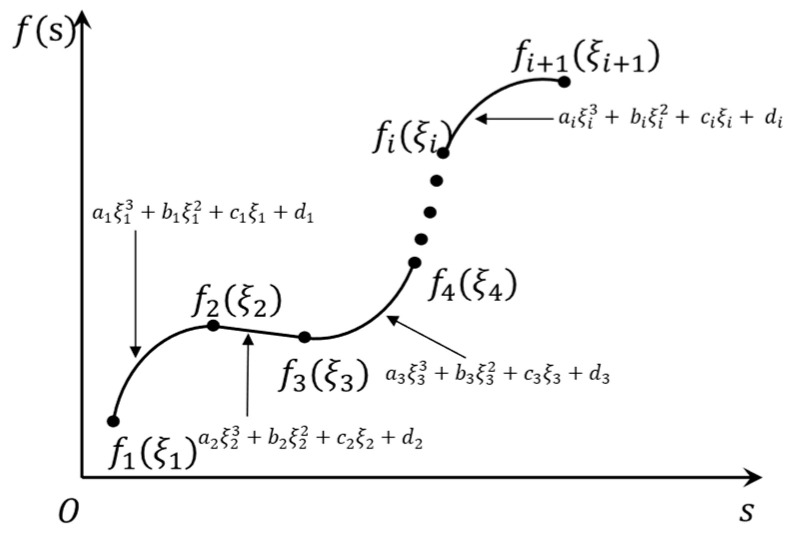
Cubic spline interpolation using the local coordinate $\xi_{i}=\;s-s_{i}$ for each spline segment.

As illustrated in Figure 1, cubic spline interpolation represents the reference path as a sequence of piecewise cubic polynomials defined over consecutive intervals. Each segment is described by a cubic function, and continuity is enforced not only in the function value but also in its first- and second-order derivatives at the connection points. This guarantees a smooth transition between segments and avoids abrupt changes in curvature.

The reference path is parameterized by the arc-length coordinate s, which allows its geometric properties to be described continuously along the track. The path orientation is represented by the reference heading angle $\psi_{r}$, while the local curvature $C_{r}$ describes the rate of change of the heading angle with respect to s. The curvature is also related to the radius of curvature $C_{r}=\frac{1}{R}$. These quantities are essential for trajectory planning and path tracking because they determine the required steering behavior and lateral motion of the vehicle. For a discrete set of path points (*X*(*s*), *Y*(*s*)), spline interpolation is used to obtain the first derivatives $X^{\prime }$ and $Y^{\prime }$, as well as the second derivatives $X^{\prime \prime }$ and $Y^{\prime \prime }$. The reference heading angle and curvature are then computed as follows:

(2) $$\psi_{r}=atan2(Y^{\prime },\;X^{\prime })$$

(3) $$C_{r}=\frac{X^{\prime }Y^{\prime \prime }-X^{\prime \prime }Y^{\prime }}{{\left[{\left(X^{\prime }\right)}^{2}+{\left(Y^{\prime }\right)}^{2}\right]}^{\frac{3}{2}}}$$

**Figure 2:**
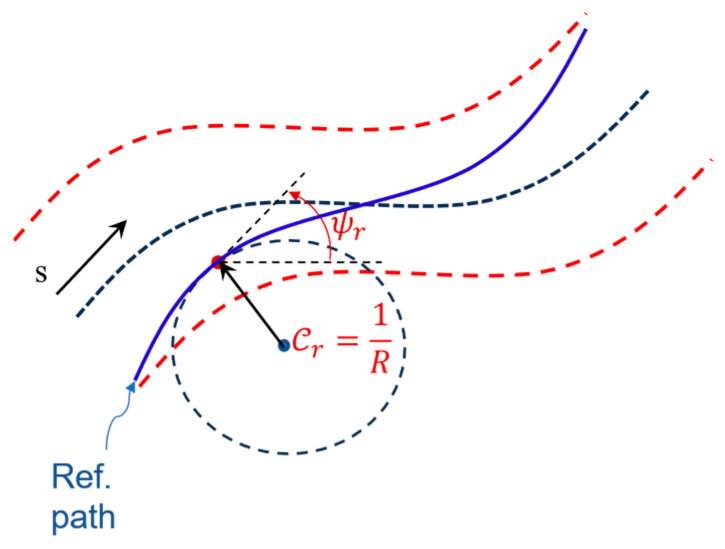
Geometric interpretation of the reference path. The blue solid curve represents the reference path, the red dashed curves denote the track boundaries, the black arrow indicates the path direction, $\psi_{r}$ is the reference heading angle, and $C_{r}=\frac{1}{R}$ is the local path curvature.

As illustrated in Figure 2, the reference path is shown together with the geometric interpretation of the heading angle and curvature. The tangent direction defines the heading angle $\psi_{r}$, while the curvature is associated with the osculating circle at a given point on the path.

**Figure 3:**
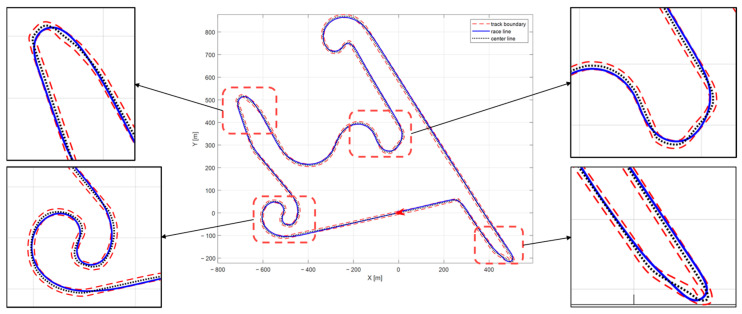
Geometric representation of the reference path and track boundaries. The blue solid line denotes the spline-fitted path, the black dashed line represents the original centerline, and the red dashed lines indicate the track boundaries.

As shown in Figure 3, the spline-fitted path (blue line) lies within the track boundaries (red dashed lines) and follows a smooth trajectory compared to the original centerline (black dashed line). This smooth reference path serves as the basis for trajectory optimization and path tracking.

**Figure 4:**
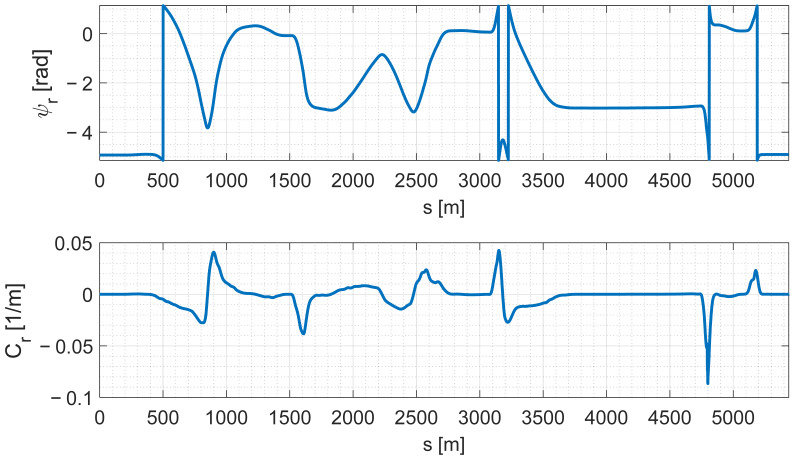
Reference heading angle and path curvature.

The corresponding profiles of $\psi_{r}$ and $C_{r}$ are shown in Figure 4. It can be observed that regions with small curvature values correspond to straight or gently curved segments, whereas larger curvature magnitudes indicate tighter corners. The apparent discontinuities in the heading angle are due to angle wrapping and do not reflect actual discontinuities in the path geometry.

## 3. Track Modeling

**Figure 5:**
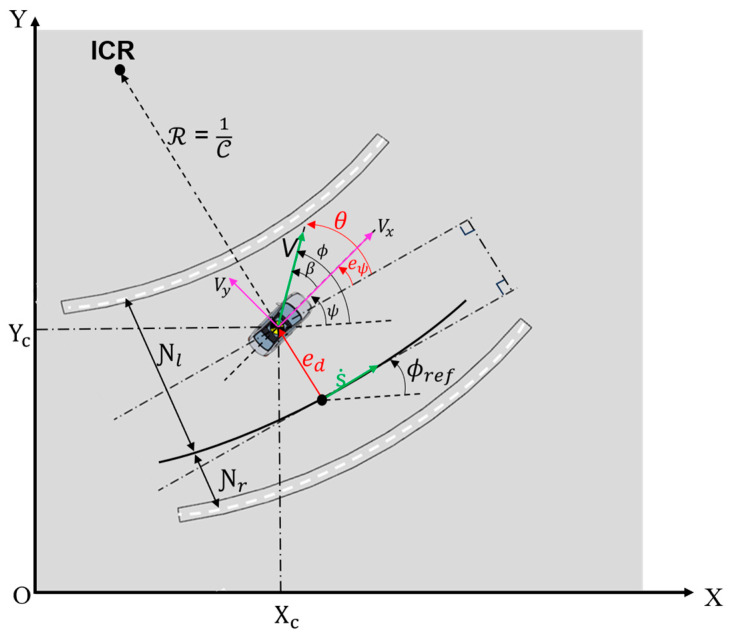
Relationship between the vehicle and the reference path kinematics. The black curve represents the reference path, the gray curves denote the track boundaries, the green arrows indicate the reference direction, and the red arrows represent the vehicle states and tracking errors.

Figure 5 illustrates the path-relative coordinate description used in this work. The course angle *ϕ* defines the direction of the vehicle velocity vector, whereas $\phi_{ref}$ denotes the tangent direction of the reference path. The vehicle position with respect to the reference path is described by the lateral deviation $e_{d}$, and the orientation mismatch is represented by the heading-angle error $e_{\psi }=\psi -\phi_{ref}$. The relative course angle is defined as $\theta =\phi -\phi_{ref}=e_{\psi }+\beta$, where β is the vehicle sideslip angle. The curvature of the reference path at the path station s is denoted by $C_{r}=\frac{d\phi_{ref}}{ds}$. The distances from the reference path to the left and right track boundaries are denoted by ${Ɲ}_{l}$ and ${Ɲ}_{r}$, respectively.

Due to the curvature of the reference path, the path-relative coordinate frame rotates with angular rate ${\dot{\phi }}_{ref}=\frac{d\phi_{ref}}{ds}\frac{ds}{dt}=C_{r}\dot{s}$. Therefore, the effective tangential velocity along the path is not simply $\dot{s}$, but is corrected by the rotational motion of the frame. The kinematic relationship is given by

(4) $$\dot{s}-e_{d}{\dot{\phi }}_{ref}=\dot{s}-e_{d}C_{r}\dot{s}=Vcos\left(\theta \right)$$

The vehicle velocity component along the tangent direction of the reference path is given by

(5) $$\dot{s}=\frac{Vcos\left(\theta \right)}{1-C_{r}\;e_{d}}=\frac{V_{x}\cos\left(e_{\psi }\right)-V_{y}\sin\left(e_{\psi }\right)}{1-C_{r}\;e_{d}}$$

The lateral deviation and heading-angle error are used as the principal path-tracking states. Based on the path-relative kinematics, their dynamic equations are expressed as

(6) $${\dot{e}}_{\psi }=\dot{\psi }-{\dot{\phi }}_{ref}=\dot{\psi }-C_{r}\dot{s}=\dot{\psi }-C_{r}\frac{V_{x}\cos\left(e_{\psi }\right)-V_{y}\sin\left(e_{\psi }\right)}{1-C_{r}\;e_{d}}$$

(7) $${\dot{e}}_{d}=V_{y}\cos\left(e_{\psi }\right)+V_{x}\sin\left(e_{\psi }\right)$$

For the TRO problem, the distance traveled along the reference path is adopted as the independent variable [17]. The elapsed time associated with each spatial grid interval can therefore be computed through the spatial scaling factor $S_{f}$, as follows:

(8) $$\frac{dt}{ds}=\frac{1-C_{r}\;e_{d}}{Vcos\left(\theta \right)}≕S_{f}$$

To guarantee that the vehicle stays inside the track boundaries, the lateral deviation is constrained by

(9) $$-{Ɲ}_{r}+\left(\frac{w_{t}}{2}+w_{s}\right)\leq e_{d}\leq {Ɲ}_{l}-\left(\frac{w_{t}}{2}+w_{s}\right)$$

where $w_{t}$ is the vehicle track width and $w_{s}=\;$0.2 m is the safety margin reserved from the track boundary. ${Ɲ}_{l}$(s) and ${Ɲ}_{r}$(s) denote the left and right track-boundary distances measured from the reference path at the path station s, respectively. These quantities are not fixed constants. They are obtained from the Shanghai International Circuit track CSV file in the racetrack database and vary along the track according to the local track geometry. The path-relative formulation is used under the assumptions of forward driving and positive vehicle speed, $V\geq V_{min}>0$. In addition, the Frenet/path-relative transformation is assumed to remain nonsingular, i.e., $1-C_{r}\;e_{d}>0$. This condition is satisfied in the considered simulations because the vehicle remains close to the reference path and inside the track-boundary constraints.

## 4. Vehicle Modeling

For racing scenarios, the vehicle model must describe the interaction among longitudinal acceleration, lateral motion, and yaw dynamics when the tires operate close to their tire–road adhesion limits. A 3-degree-of-freedom double-track model is therefore adopted in both the offline trajectory optimization and the online NMPC controller. Aerodynamic drag and lift are included because they influence the achievable speed and tire normal loads at high velocity. Suspension dynamics are not modeled in order to keep the optimization problem tractable. The simulated platform is a four-wheel-drive sports car with four independent motors, and the main vehicle parameters are listed in Table A1.

### 4.1. Double-Track Vehicle Model

**Figure 6:**
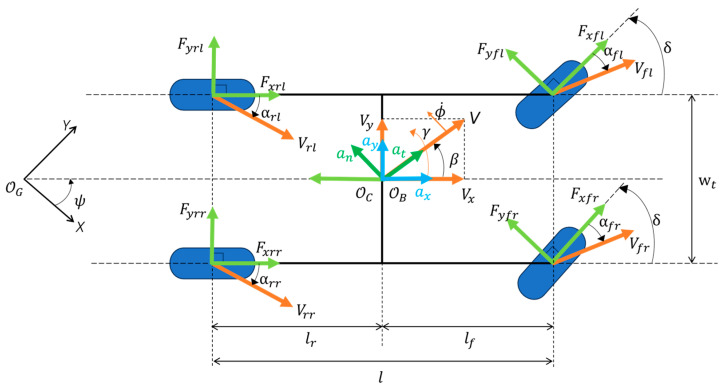
Coordinate systems, vehicle geometry, and tire-force definitions. Blue, green, and orange arrows denote body-fixed velocities, path-aligned accelerations, and tire longitudinal velocities, respectively.

The vehicle dynamics are modeled using a double-track representation, as illustrated in Figure 6. The vehicle heading is defined by the yaw angle *ψ* with respect to the X-axis of the global coordinate system $O_{G}$. The longitudinal and lateral accelerations $a_{x}$ and $a_{y}$ are expressed in the body-fixed frame $O_{B}$, while $a_{t}$ and $a_{n}$ are defined in the path-aligned coordinate system $O_{C}$.

The vehicle acceleration components can be written as

(10) $$a_{x}=\frac{1}{m}\left(F_{xf}cos\left(\delta \right)-F_{yf}sin\left(\delta \right)+F_{xr}-F_{drag}\right),$$

(11) $$a_{y}=\frac{1}{m}\left(F_{yf}cos\left(\delta \right)+F_{xf}\sin(\delta )+F_{yr}\right),$$

(12) $$a_{t}=\dot{V}=\frac{1}{m}\left(F_{xf}cos\left(\delta -\beta \right)-F_{yf}sin\left(\delta -\beta \right)+F_{xr}cos\left(\beta \right)+F_{yr}sin\left(\beta \right)-F_{drag}cos\left(\beta \right)\right),$$

(13) $$a_{n}=\frac{1}{m}\left(F_{xf}sin\left(\delta -\beta \right)+F_{yf}cos\left(\delta -\beta \right)-F_{xr}sin\left(\beta \right)+F_{yr}cos\left(\beta \right)+F_{drag}sin\left(\beta \right)\right),$$

where m denotes the vehicle mass, δ is the front-wheel steering angle, and $F_{drag}$ = $\frac{1}{2}\rho C_{d}AV_{x}^{2}$ represents the aerodynamic drag force. The parameters ρ, $C_{d}$, and A represent the air density, drag coefficient, and frontal area, respectively. $V_{x}$ is the longitudinal velocity. The axle-level tire forces are obtained by summing the left and right wheel forces, namely $F_{xf}=F_{xfl}+\;F_{xfr}$, $F_{xr}=F_{xrl}+\;F_{xrr}$, $F_{yf}=F_{yfl}+\;F_{yfr}$, $F_{yr}=F_{yrl}+\;F_{yrr}$.

The yaw moment about the vehicle center of gravity (CG) can be expressed as

(14) $$\begin{array}{l} M_{z}=l_{f}F_{yf}cos\left(\delta \right)+l_{f}F_{xf}sin\left(\delta \right)-l_{r}F_{yr}\\ \;\;\;\;\;\;\;\;\;\;+\frac{w_{t}}{2}\left[(F_{yfl}-F_{yfr})sin\left(\delta \right)+(F_{xfr}-F_{xfl})cos\left(\delta \right)+(F_{xrr}-F_{xrl})\right] \end{array}$$

where $l_{f}$ and $l_{r}$ are the distances from the vehicle CG to the front and rear axles, respectively.

Using the tangential acceleration, sideslip-angle dynamics, and yaw moment balance, the planar vehicle dynamics are written as

(15) $$\dot{V}=a_{t}$$

(16) $$\dot{\beta }=\dot{\phi }-\dot{\psi }=\frac{a_{n}}{V}-\gamma$$

(17) $$\dot{\gamma }=\frac{M_{z}}{I_{z}}$$

where $I_{z}$ denotes the yaw moment of inertia of the vehicle.

Because the sideslip-angle dynamics contain the term $\frac{a_{n}}{V}$, a positive lower bound on the vehicle speed is required. Therefore, the vehicle speed is constrained as $V\geq V_{min}>0$ in both the TRO and NMPC formulations.

### 4.2. Load Transfer

To account for tire force variations caused by significant longitudinal and lateral accelerations, a load transfer model based on [18] is adopted:

(18) $$F_{zfl}=\frac{1}{2}m\left(g\frac{l_{r}}{l}-{\bar{a}}_{x}\frac{h}{l}\right)-m\left(g\frac{l_{r}}{l}-{\bar{a}}_{x}\frac{h}{l}\right)\frac{{\bar{a}}_{y}}{g}\frac{h}{w_{t}}-\frac{F_{lift}}{4}$$

(19) $$F_{zfr}=\frac{1}{2}m\left(g\frac{l_{r}}{l}-{\bar{a}}_{x}\frac{h}{l}\right)+m\left(g\frac{l_{r}}{l}-{\bar{a}}_{x}\frac{h}{l}\right)\frac{{\bar{a}}_{y}}{g}\frac{h}{w_{t}}-\frac{F_{lift}}{4}$$

(20) $$F_{zrl}=\frac{1}{2}m\left(g\frac{l_{f}}{l}+{\bar{a}}_{x}\frac{h}{l}\right)-m\left(g\frac{l_{f}}{l}+{\bar{a}}_{x}\frac{h}{l}\right)\frac{{\bar{a}}_{y}}{g}\frac{h}{w_{t}}-\frac{F_{lift}}{4}$$

(21) $$F_{zrr}=\frac{1}{2}m\left(g\frac{l_{f}}{l}+{\bar{a}}_{x}\frac{h}{l}\right)+m\left(g\frac{l_{f}}{l}+{\bar{a}}_{x}\frac{h}{l}\right)\frac{{\bar{a}}_{y}}{g}\frac{h}{w_{t}}-\frac{F_{lift}}{4}$$

where l, h, and *g* represent the wheelbase, the CG height, and gravitational acceleration, respectively. The quantities ${\bar{a}}_{x}$ and ${\bar{a}}_{y}$ are treated as decision variables in the nonlinear programming (NLP) problem. The aerodynamic lift force is expressed as $F_{lift}=\frac{1}{2}\rho C_{l}AV_{x}^{2}$, with $C_{l}$ being the lift coefficient.

The auxiliary variables ${\bar{a}}_{x}$ and ${\bar{a}}_{y}$ are introduced to avoid direct algebraic coupling during optimization. Their values are constrained to match the longitudinal and lateral accelerations computed from the vehicle dynamics:

(22) $${\bar{a}}_{x}-a_{x}=0,$$

(23) $${\bar{a}}_{y}-a_{y}=0.$$

For the online NMPC formulation, these acceleration consistency constraints are approximated by first-order dynamics to improve numerical convergence:

(24) $${\dot{\bar{a}}}_{x}=\frac{1}{\tau }\left(a_{x}-{\bar{a}}_{x}\right)$$

(25) $${\dot{\bar{a}}}_{y}=\frac{1}{\tau }\left(a_{y}-{\bar{a}}_{y}\right)$$

where the time constant τ is chosen as half of the NMPC sampling time.

The first-order acceleration dynamics in the NMPC formulation are introduced for numerical rather than physical reasons. They avoid direct algebraic coupling between the load-transfer model, tire-force calculation, and vehicle acceleration equations, thereby improving the conditioning of the online optimization problem. The time constant is selected as one half of the NMPC sampling time, allowing the filtered accelerations to rapidly follow the acceleration values computed from the vehicle dynamics. Therefore, this filtered acceleration model should be interpreted as a real-time-oriented approximation that provides a compromise between numerical robustness and physical fidelity.

### 4.3. Wheel Torques

**Figure 7:**
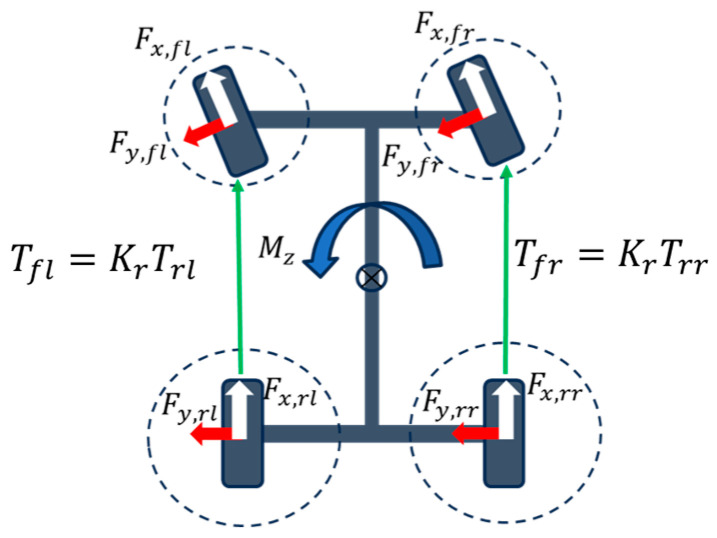
Schematic of the rear-dominant virtual 4WD torque distribution strategy. The red arrows denote the tire longitudinal forces, the green dashed lines represent the virtual front–rear torque coupling, and the blue curved arrow indicates the generated yaw moment $M_{z}$.

Figure 7 illustrates the proposed rear-dominant four-wheel-drive torque distribution strategy. To exploit the traction capability of the four-wheel-drive vehicle while maintaining a reduced-complexity control structure, a virtual 4WD torque distribution strategy is adopted. The NMPC controller optimizes only the two rear-wheel driving torques $T_{rr}$ and $T_{rl}$, while the front-wheel torques are generated through a gain-based distribution law. As $K_{r}$ increases, the front tire longitudinal force also increases, which improves acceleration capability but reduces the available lateral tire force due to the tire–road adhesion ellipse constraint.

The front-wheel torques are defined as

(26) $$T_{fl}=K_{r}T_{rl},$$

(27) $$T_{fr}=K_{r}T_{rr},$$

where $K_{r}$ denotes the proportional front-to-rear torque distribution gain.

To preserve the rear-dominant behavior of the proposed virtual 4WD strategy, the nominal design range of ($K_{r}$) is selected as

(28) $$0\leq K_{r}\leq 0.5$$

In this study, $K_{r}$ was selected empirically based on closed-loop sensitivity simulations and should therefore be interpreted as a heuristic design parameter. Adaptive or optimization-based selection of $K_{r}$ will be considered in future work to improve robustness under varying tire-load and adhesion conditions.

Additional larger values are evaluated only to examine the degradation mechanism outside the nominal design range.

This constraint guarantees that the front-wheel driving torque does not exceed the corresponding rear-wheel torque.

### 4.4. Tire Modeling

For each wheel, the rotational motion is governed by the balance between the applied wheel torque and the resistive moment generated by the longitudinal tire force. The wheel-speed dynamics are therefore expressed as follows:

(29) $${\dot{\omega }}_{fl}=\frac{T_{fl}-RF_{x_{fl}}}{I_{w}}$$

(30) $${\dot{\omega }}_{fr}=\frac{T_{fr}-RF_{x_{fr}}}{I_{w}}$$

(31) $${\dot{\omega }}_{rl}=\frac{T_{rl}-RF_{x_{rl}}}{I_{w}}$$

(32) $${\dot{\omega }}_{rr}=\frac{T_{rr}-RF_{x_{rr}}}{I_{w}}$$

where $T_{fl}$, $T_{fr}$, $T_{rl}$, and $T_{rr}$ are the torques applied to the corresponding wheels, $I_{w}$ is the wheel rotational inertia, and R represents the effective rolling radius.

The longitudinal velocity components at the tire contact points are calculated by considering the vehicle longitudinal velocity, yaw motion, track width, and front steering angle:

(33) $$V_{x,fl}\;=\;(V_{x}-\frac{w_{t}}{2}\gamma )\cos\left(\delta \right)\;+\;(V_{y}+l_{f}\gamma )\sin\left(\delta \right)$$

(34) $$V_{x,fr}=(V_{x}+\frac{w_{t}}{2}\gamma )\cos\left(\delta \right)+\;(V_{y}+l_{f}\gamma )\sin\left(\delta \right)$$

(35) $$V_{x,rl}=V_{x}-\frac{w_{t}}{2}\gamma \;$$

(36) $$V_{x,rr}=V_{x}+\frac{w_{t}}{2}\gamma \;$$

Similarly, the lateral velocity components at each tire are obtained from the vehicle lateral velocity, yaw-rate contribution, and steering transformation for the front wheels:

(37) $$V_{y,fl}\;=\;(V_{y}+l_{f}\gamma )\cos\left(\delta \right)-(V_{x}-\frac{w_{t}}{2}\gamma )\sin\left(\delta \right)$$

(38) $$V_{y,fr}=(V_{y}+l_{f}\gamma )\cos\left(\delta \right)-(V_{x}+\frac{w_{t}}{2}\gamma )\sin\left(\delta \right)$$

(39) $$V_{y,rl}=V_{y}-l_{r}\gamma$$

(40) $$V_{y,rr}=V_{y}-l_{r}\gamma$$

Then, the longitudinal slip λ and sideslip angle α of the tire at each corner i ∈ {*fl*, *fr*, *rl*, *rr*} are defined by

(41) $$\lambda_{i}=\frac{{R\omega }_{i}-V_{xi}}{V_{xi}}$$

(42) $$\alpha_{i}={tan}^{-1}\left(\frac{V_{yi}}{V_{xi}}\right)$$

### 4.5. Simplified MF Tire Model

**Figure 8:**
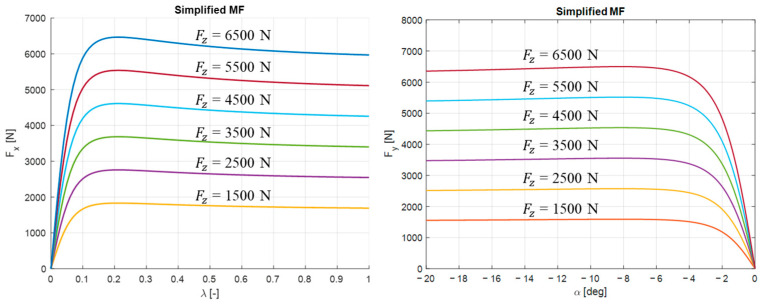
Steady-state Magic Formula tire characteristics. The side-slip angle is shown with its signed value according to the vehicle-model convention; the negative sign indicates the assumed tire side-slip direction.

The simplified Magic Formula tire model used in this study represents the steady-state relationship between tire slip and tire force [19]. This choice provides a differentiable and computationally efficient tire-force model for the gradient-based TRO and NMPC optimization problems. Under pure longitudinal slip, the tire force is calculated from the slip ratio λ, the vertical tire load $F_{z}$, and the road–tire adhesion coefficient μ, as shown in the left plot of Figure 8. Since transient tire relaxation is not explicitly modeled, the tire-force prediction should be interpreted based on a steady-state tire-force assumption. Non-steady-state tire models will be considered in future work to improve the prediction of transient lateral tire dynamics.

(43) $$F_{x0}\left(\lambda ,F_{z},\mu \right)=\frac{\mu }{\mu_{0}}D_{x}\sin\left[C_{x}{tan}^{-1}\left(B_{x}\lambda \right)\right]$$

where $\mu_{0}$ is the nominal adhesion coefficient used for tire-parameter identification. The coefficients $B_{x}$, $C_{x}$, and $D_{x}$ correspond to the stiffness, shape, and peak factors of the longitudinal tire model, respectively. Because the peak force changes with the vertical load, $D_{x}$ is represented by a linear function of $F_{z}$:

(44) $$D_{x}=\;d_{1x}F_{z}+d_{2x}$$

where $d_{1x}$ and $d_{2x}$ are the load-dependent slope and constant offset, respectively.

The lateral force under pure sideslip is obtained in a similar manner. As illustrated in the right plot of Figure 8, the lateral tire force is computed from the sideslip angle *α*, vertical load $F_{z}$, and adhesion coefficient μ:

(45) $$F_{y0}\left(\alpha ,F_{z},\mu \right)=-\frac{\mu }{\mu_{0}}D_{y}\sin\left[C_{y}{tan}^{-1}\left(B_{y}\alpha \right)\right]$$

The parameters $B_{y}$, $C_{y}$, and $D_{y}$ define the lateral stiffness, shape, and peak factors, respectively. The lateral peak factor is given by $D_{y}$ = $d_{1y}F_{z}+d_{2y}$, where $d_{1y}$ and $d_{2y}$ are the corresponding load-dependent coefficients.

In Figure 8, the side-slip angle is plotted with its signed value according to the vehicle-model convention. The negative sign only indicates the assumed tire side-slip direction and does not imply an abnormal tire characteristic. The signed side-slip angle is retained in the simulation because it determines the direction of the lateral tire force and is required for consistency with the lateral-yaw vehicle dynamics.

### 4.6. Combined Slip Tire Model for TRO

During aggressive cornering, the tires are often required to generate longitudinal and lateral forces simultaneously. This produces a coupled-slip condition that should be considered when generating a near-limit reference trajectory.

Therefore, the TRO formulation uses a combined-slip tire model (ch. 4.2.2, [19]) to obtain a more physically feasible racing trajectory. To avoid numerical singularity when the resultant slip approaches zero, the regularized resultant slip magnitude at each tire is defined as

(46) $$\sigma_{\varepsilon ,\;\;i}=\sqrt{\lambda_{i}^{2}+{tan}^{2}\left(\alpha_{i}\right)+\varepsilon_{\sigma }^{2}},i\in \left\{fl,fr,rl,rr\right\}$$

where $\varepsilon_{\sigma }>0$ is a small positive regularization constant.

The longitudinal and lateral tire forces are then obtained by projecting the pure-slip tire-force response along the corresponding slip directions:

(47) $$F_{xi}=\frac{\lambda_{i}}{\sigma_{\varepsilon ,\;\;i}}F_{x0}\left(\sigma_{\varepsilon ,i},F_{zi},\mu \right),\;\;\;\;i\in \left\{fl,fr,rl,rr\right\}$$

(48) $$F_{yi}=\frac{tan\left(\alpha_{i}\right)}{\sigma_{\varepsilon ,\;\;i}}F_{y0}\left(\sigma_{\varepsilon ,i},F_{zi},\mu \right),\;\;\;i\in \left\{fl,fr,rl,rr\right\}$$

This regularization prevents division by zero when both the longitudinal slip and tire sideslip angle approach zero.

### 4.7. Pure Sideslip Tire Model for NMPC

For the online NMPC problem, the prediction model is simplified to improve computational efficiency. Wheel rotational dynamics and the full combined-slip model are not included explicitly. Instead, the longitudinal force is calculated directly from the wheel torque command, and the lateral force is obtained from the pure sideslip tire model. The coupling between longitudinal and lateral forces is enforced separately through the tire–road adhesion ellipse constraints:

(49) $$F_{xi}=\frac{T_{i}}{R},\;i\in \left\{fl,fr,rl,rr\right\}$$

(50) $$F_{yi}=F_{y0}\left(\alpha_{i},F_{zi},\mu \right),\;i\in \left\{fl,fr,rl,rr\right\}$$

The offline TRO and online NMPC use tire models with different levels of fidelity. The TRO adopts the combined-slip Magic Formula tire model because it is solved offline and is used to generate a near-limit racing trajectory. In contrast, the online NMPC uses a reduced tire model to reduce computational burden and improve real-time feasibility. In the NMPC model, the longitudinal tire force is computed from the wheel torque command, while the lateral tire force is obtained from the pure sideslip tire model. Although the full combined-slip tire model is not included in the NMPC prediction model, the combined longitudinal–lateral tire-force demand is still considered through the tire–road adhesion ellipse constraint. Therefore, the reduced NMPC tire model is used as a real-time-oriented approximation, while the adhesion ellipse constraint is retained to limit the combined tire-force demand during near-limit operation.

### 4.8. Vehicle Constraints

The tire-force operating region is restricted by quadratic tire–road adhesion ellipse constraints. These constraints prevent the combined longitudinal and lateral tire forces from exceeding the available tire–road adhesion potential:

(51) $${\left(\frac{F_{xi}}{\mu_{x,max}F_{zi}}\right)}^{2}+{\left(\frac{F_{yi}}{\mu_{y,max}F_{zi}}\right)}^{2}\leq 1,\;i\in \left\{fl,fr,rl,rr\right\}$$

where $\mu_{x,max}$ and $\mu_{y,max}$ are the longitudinal and lateral adhesion limits.

Because the vehicle has four independent electric motors, the power of each wheel motor is constrained separately in both driving and braking modes. In the TRO formulation, the wheel angular velocity $\omega_{i}$ is directly included as a state variable, and the motor-power constraint is written in the general form:

(52) $$-P_{i,brake,max}\leq T_{i}\omega_{i}\leq P_{i,drive,max},\;i\in \left\{fl,fr,rl,rr\right\}$$

In the NMPC formulation, the wheel rotational dynamics are not included as prediction states. Therefore, the wheel angular velocity is approximated by $\omega_{i}\approx \frac{V_{x,i}}{R}$. After substituting the virtual 4WD torque distribution law, the NMPC motor-power constraints become

(53) $$-P_{fl,brake,max}\leq K_{r}T_{rl}\frac{V_{x,fl}}{R}\leq P_{fl,drive,max}$$

(54) $$-P_{fr,brake,max}\leq K_{r}T_{rr}\frac{V_{x,fr}}{R}\leq P_{fr,drive,max}$$

(55) $$-P_{rl,brake,max}\leq T_{rl}\frac{V_{x,rl}}{R}\leq P_{rl,drive,max}$$

(56) $$-P_{rr,brake,max}\leq T_{rr}\frac{V_{x,rr}}{R}\leq P_{rr,drive,max}$$

where $P_{i,drive,max}$ and $P_{i,brake,max}$ denote the maximum driving and braking power limits of wheel *i*, respectively. $V_{x,i}$ is the longitudinal velocity at the corresponding tire contact point, and R is the effective rolling radius, with i ∈{fl, fr, rl, rr}. In the NMPC formulation, the wheel-speed approximation $\omega_{i}\approx \frac{V_{x,i}}{R}$ is adopted to reduce the prediction-model complexity. This approximation is valid under moderate longitudinal-slip conditions, where the wheel circumferential speed remains close to the longitudinal velocity at the tire contact point. The resulting tire-force demand is still bounded by the tire–road adhesion ellipse constraints.

## 5. Control Architecture

**Figure 9:**
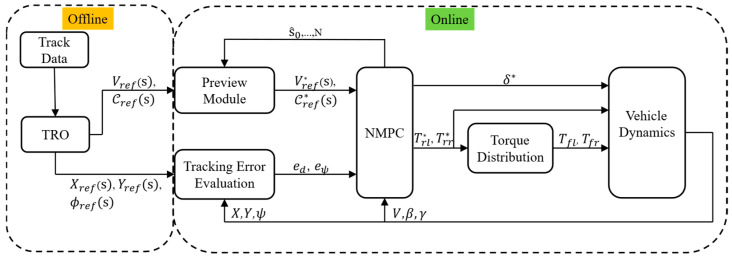
Overall offline–online control framework integrating TRO, NMPC, and virtual 4WD torque distribution. The yellow region represents the offline trajectory optimization process, while the green region represents the online control process.

The overall control framework consists of offline and online components, as illustrated in Figure 9. The offline trajectory optimization (TRO) module computes a minimum-time trajectory using the provided track data. The resulting reference race-line, velocity profile, and curvature are then passed to the online stage. The tracking error module computes the lateral deviation and heading error during online path tracking using the method proposed in [20]. The preview module determines the corresponding speed profiles and reference curvature based on the previewed path station. The NMPC module computes the steering input and the rear-left and rear-right wheel torque commands. The front-wheel torques are then generated through the proposed gain-based virtual 4WD torque distribution strategy. The resulting wheel torques are applied to the vehicle model. In the following iteration, the measured vehicle states are fed back to the controller for closed-loop optimization.

## 6. Trajectory Optimization

This section presents the offline trajectory optimization procedure used to obtain the minimum-lap-time reference trajectory for the racetrack. The optimization simultaneously computes the vehicle state trajectory and the corresponding control inputs while enforcing vehicle dynamics, actuator limits, tire-force constraints, and track-boundary restrictions. Before the optimization is performed, the racetrack centerline is processed to obtain a smooth spatial representation and is divided into uniformly spaced grid points. The continuous optimal control problem is then converted into a nonlinear programming (NLP) problem using direct orthogonal collocation in the spatial domain.

### 6.1. System Dynamics

The TRO problem is solved in the spatial domain by selecting the path station s as the independent variable instead of time. The optimization state is defined to include the main vehicle dynamic variables, the wheel angular velocities, and the path-tracking error states:

(57) $$x={\left[\begin{array}{ccccccccc} V & \beta & \gamma & \omega_{fl} & \omega_{fr} & \omega_{rl} & \omega_{rr} & e_{d} & e_{\psi } \end{array}\right]}^{T}$$

where $\omega_{fl}$, $\omega_{fr}$, $\omega_{rl}$, and $\omega_{rr}$ denote the wheel angular velocities and $e_{d}$ and $e_{\psi }$ represent the lateral deviation and heading-angle error, respectively.

The control vector consists of the front steering angle and the rear-left and rear-right driving/braking torques:

(58) $$u={\left[\begin{array}{ccc} T_{rl} & T_{rr} & \delta \end{array}\right]}^{T}$$

The filtered longitudinal and lateral accelerations are introduced as auxiliary variables:

(59) $$z={\left[\begin{array}{cc} {\bar{a}}_{x} & {\bar{a}}_{y} \end{array}\right]}^{T}$$

The system dynamics are then expressed as a function of the state, control, auxiliary, and parameter variables:

(60) $$\begin{array}{rl} \dot{x} & ={\left[\begin{array}{ccccccccc} \dot{V} & \dot{\beta } & \dot{\gamma } & {\dot{\omega }}_{fl} & {\dot{\omega }}_{fr} & {\dot{\omega }}_{rl} & {\dot{\omega }}_{rr} & {\dot{e}}_{d} & {\dot{e}}_{\psi } \end{array}\right]}^{T}\\ & ={\left[\begin{array}{ccccccccc} \left(15\right) & \left(16\right) & \left(17\right) & \left(29\right) & \left(30\right) & \left(31\right) & \left(32\right) & \left(7\right) & \left(6\right) \end{array}\right]}^{T} \end{array}$$

The time-domain model is converted into a spatial-domain model through the scaling factor $S_{f}$:

(61) $$x^{\prime }=\frac{dx}{ds}=\frac{dt}{ds}\frac{dx}{dt}=S_{f}\dot{x}≕f_{s}(x,u,z,C)$$

The state, control, and auxiliary variables are restricted by their physical lower and upper limits:

(62) $$x_{min}\leq x\leq x_{max}$$

(63) $$u_{min}\leq u\leq u_{max}$$

(64) $$z_{min}\leq z\leq z_{max}$$

Here, the subscripts min and max indicate the admissible lower and upper bounds.

In addition, actuator bandwidth limitations are represented by imposing bounds on the time derivatives of the control inputs:

(65) $${\dot{u}}_{min}\leq \dot{u}\leq {\dot{u}}_{max}$$

### 6.2. Direct Collocation Method

**Figure 10:**
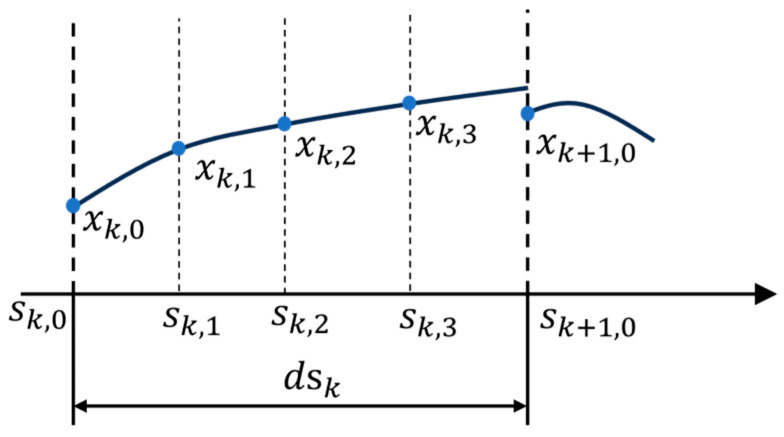
The direct collocation with *q* = 3 in a segment ${ds}_{k}$.

Figure 10 illustrates the spatial discretization used in the direct collocation formulation. The track coordinate is divided into N intervals, where each segment spans [$s_{k}$,$s_{k+1}$] with length ${ds}_{k}$, for k=0,…,N−1.

Within each spatial interval, the state trajectory is approximated by a polynomial interpolation function based on Lagrange basis polynomials:

(66) $$x_{k}\left(\tau \right)=\sum_{i=0}^{q}P_{i}\left(\tau \right)x_{k,i},\;i=0,\ldots ,q$$

where $P_{i}\left(\tau \right)$ denotes the Lagrange basis polynomial associated with the Legendre collocation points over the normalized interval τ ∈ [0, 1]. The basis polynomial is constructed using the collocation points {$\tau_{0}$, $\tau_{1}$, …, $\tau_{q}$} (ch. 10.3, [21]) with order q, as follows:

(67) $$P_{i}\left(\tau \right)=\prod_{\begin{array}{c} j=0,\\ j\neq i \end{array}}^{q}\frac{\tau -\tau_{j}}{\tau_{i}-\tau_{j}},\;i=0,\ldots ,q$$

To express the polynomial state approximation in a compact form, the state values at the initial node and at the collocation points of the k interval are assembled into the matrix $X_{k}$:

(68) $$X_{k}=\left[\begin{array}{cccc} x_{k,0} & x_{k,1} & \ldots & x_{k,q} \end{array}\right]\in R^{n_{x}\times \left(q+1\right)},\;k=0,\ldots ,N-1.$$

The direct collocation method is then used to transcribe the TRO problem into a finite-dimensional NLP [22,23,24]. In this approach, the polynomial approximation is constrained to satisfy the spatial-domain system dynamics at each collocation point.

For each interval, the derivative of the interpolating polynomial evaluated at the collocation points is enforced to match the spatial-domain dynamics:

(69) $$X_{k}C-{ds}_{k}\left[\begin{array}{ccc} x_{k,1}^{\prime } & \ldots & x_{k,q}^{\prime } \end{array}\right]=0,\;k=0,\ldots ,N-1,$$

where $x_{k,j}^{\prime }$ = $f_{s}$($x_{k,j},u_{k},\;z_{k},C_{k}$) denotes the spatial-domain vehicle dynamics at the collocation points, and the control input $u_{k}$ is assumed to be piecewise constant over the interval [$s_{k}$,$s_{k+1}$).

In addition, a continuity constraint is imposed to ensure that the terminal state of the current interval is consistent with the initial state of the next interval:

(70) $$X_{k}D-x_{k+1}=0,\;k=0,\ldots ,N-1,$$

The coefficient matrix ***C*** collects the derivatives of the Lagrange basis functions at the collocation points, whereas ***D*** contains the corresponding basis-function values evaluated at the terminal point of the interval:

(71) $$C=\left[\begin{array}{ccc} {\dot{P}}_{0}\left(\tau_{1}\right) & \ldots & {\dot{P}}_{0}\left(\tau_{q}\right)\\ \vdots & \ddots & \vdots \\ {\dot{P}}_{q}\left(\tau_{1}\right) & \ldots & {\dot{P}}_{q}\left(\tau_{q}\right) \end{array}\right]\in R^{\left(q+1\right)\times q},\;\;\;D=\left[\begin{array}{c} P_{0}\left(1\right)\\ \vdots \\ P_{q}\left(1\right) \end{array}\right]\in R^{\left(q+1\right)}.$$

Additional constraints, denoted by h, are imposed at the knot points to account for the tire–road adhesion ellipse, actuator limits, motor power limits, and state/control constraints:

(72) $$h_{min}\leq h\left(x_{k},u_{k},z_{k},C_{k}\right)\leq h_{max},\;k=0,\ldots ,N$$

where $h_{min}$ and $h_{max}$ represent the lower and upper limits of the constraint vector, respectively.

Since the TRO problem is formulated in the spatial domain, the variation of the control inputs with respect to distance is converted into the corresponding time-domain rate using the path velocity $\dot{s}$. This allows the actuator rate limits to be enforced consistently in the time domain:

(73) $$\dot{u}\approx \frac{du}{ds}\frac{ds}{dt}=u^{\prime }\dot{s}\to {\dot{u}}_{min}\leq u^{\prime }\dot{s}\leq {\dot{u}}_{max}$$

### 6.3. TRO Cost Function

The cost function defines the optimization objective of the TRO problem. Since the main objective is to minimize the lap time, the time required to travel through each discretized path interval is included in the cost function. The time increment of the interval is computed as

(74) $${dt}_{k}={ds}_{k}\left[\begin{array}{ccc} S_{f}\left(x_{k,1},C_{k}\right) & \ldots & S_{f}\left(x_{k,q},C_{k}\right) \end{array}\right]B,\;k=0,\ldots ,N-1$$

where the matrix B contains the quadrature weights associated with the collocation points and is used to approximate the time integral within each interval:

(75) $$B\;=\;{\left[\begin{array}{ccc} \int_{0}^{1}P_{1}\left(\tau \right)d\tau & \ldots & \int_{0}^{1}P_{q}\left(\tau \right)d\tau \end{array}\right]}^{T}\in R^{q}$$

To obtain a minimum-lap-time trajectory while maintaining smooth control inputs, the overall TRO cost function is formulated as the sum of the travel-time cost and quadratic penalties on the spatial variations of the control and auxiliary variables:

(76) $$J_{TRO}=\sum_{k=0}^{N-1}\left({dt}_{k}+{u_{k}^{\prime }}^{T}Ru_{k}^{\prime }+{z_{k}^{\prime }}^{T}Wz_{k}^{\prime }\right)$$

where R and W are weighting matrices for penalizing the variations of the control inputs and auxiliary variables, respectively. Larger weights can be used to suppress oscillatory behavior in the optimized input trajectories. The spatial variations of the control and auxiliary variables are approximated by finite differences:

(77) $$u_{k}^{\prime }=\frac{u_{k+1}-u_{k}}{{ds}_{k}},\;z_{k}^{\prime }=\frac{z_{k+1}-z_{k}}{{ds}_{k}},\;\;\;\;k=0,\ldots ,N-1$$

### 6.4. NLP Solver

To improve the numerical convergence of the NLP solver, the decision variables are properly scaled before optimization [25]. The state, control, and auxiliary variables are normalized using scaling factors determined from their expected maximum values. This scaling procedure keeps the decision variables within a comparable numerical range, typically between −1 and 1, thereby improving the conditioning of the optimization problem. Details of the scaling strategy and its implementation can be found in [26].

The overall TRO formulation is summarized in Table 1, including the decision variables, constraints, lower and upper bounds, and scaling factors. The TRO problem is formulated symbolically using CasADi [27] and transcribed into a nonlinear programming (NLP) problem. The resulting large-scale NLP is solved using the Ipopt nonlinear optimization solver [28]. The main TRO optimization parameters, bounds, weighting matrices, and solver settings are summarized in Table A2.

## 7. Nonlinear Model Predictive Control

In the proposed architecture, the NMPC serves as the online closed-loop tracking controller. At each sampling instant, it computes the rear-wheel torque-rate commands and steering-rate command required to follow the TRO-generated reference while satisfying vehicle, actuator, tire, and track constraints.

### 7.1. Prediction Model

To predict the future vehicle motion, the NMPC prediction model includes the vehicle dynamic states, path station, path-tracking errors, and the integrated control commands. The state vector is defined as

(78) $$x={\left[\begin{array}{ccccccccccc} V & \beta & \gamma & {\bar{a}}_{x} & {\bar{a}}_{y} & s & e_{d} & e_{\psi } & T_{rl} & T_{rr} & \delta \end{array}\right]}^{T}$$

The control inputs are selected as the rates of the rear-wheel torque commands and the steering input:

(79) $$u={\left[\begin{array}{ccc} {\dot{T}}_{rl} & {\dot{T}}_{rr} & \dot{\delta } \end{array}\right]}^{T}$$

The prediction model dynamics are formulated as

(80) $$\begin{array}{rl} \dot{x} & ={\left[\begin{array}{ccccccccccc} \dot{V} & \dot{\beta } & \dot{\gamma } & {\dot{\bar{a}}}_{x} & {\dot{\bar{a}}}_{y} & \dot{s} & {\dot{e}}_{d} & {\dot{e}}_{\psi } & {\dot{T}}_{rl} & {\dot{T}}_{rr} & \dot{\delta } \end{array}\right]}^{T}\\ & ={\left[\begin{array}{ccccccccccc} \left(15\right) & \left(16\right) & \left(17\right) & \left(24\right) & \left(25\right) & \left(5\right) & \left(7\right) & \left(6\right) & {\dot{T}}_{rl} & {\dot{T}}_{rr} & \dot{\delta } \end{array}\right]}^{T}\\ & ≕f_{p}(x,u,C) \end{array}$$

The first five components of (78) describe the vehicle speed, sideslip angle, yaw rate, and filtered longitudinal and lateral acceleration states. The next three components predict the path station, lateral deviation, and heading-angle error. The last three components integrate the rear-wheel torque rates and steering-rate input, resulting in smooth rear-wheel torque and steering commands. This rate-based input formulation is similar to those used in [1,29,30] and allows the control variations to be directly penalized in the NMPC cost function, consistent with the TRO formulation in (76).

The state and control variables are bounded according to the physical limitations of the vehicle and actuators:

(81) $$x_{min}\leq x\leq x_{max}$$

(82) $$u_{min}\leq u\leq u_{max}$$

### 7.2. Discretization

The prediction model is discretized using the implicit trapezoidal scheme, which is a second-order implicit Runge–Kutta method. The initial state of the prediction horizon is set to the measured or estimated vehicle state at the current sampling instant:

(83) $$x_{0}=\hat{x}\left(t\right)$$

(84) $$x_{k+1}=x_{k}+\frac{t_{s}}{2}\left[f_{p}\left(x_{k},u_{k},C_{k}\right)+f_{p}\left(x_{k+1},u_{k+1},C_{k+1}\right)\right],\;k=0,\ldots ,\;N_{p}-1$$

where $\hat{x}\left(t\right)$ denotes the current state estimate. The NMPC sampling time is set to $t_{s}$ = 0.05 s, and the prediction horizon is chosen as $N_{p}$ = 30, resulting in a total prediction time of $T_{p}$ = $N_{p}t_{s}$ = 1.5 s.

### 7.3. Preview Path Station

To generate the reference trajectory for the NMPC prediction horizon, a preview path-station update strategy is adopted. The preview path stations are obtained by shifting the optimal path-station sequence from the previous NMPC solution forward by one step. Specifically, the preview path station at stage k is defined as

(85) $${\hat{s}}_{k}=s_{k+1}^{*},\;k=0,\ldots ,N_{p}-1,$$

This shifting strategy allows the NMPC controller to use the most recently optimized path-station sequence as the reference for the next sampling instant.

**Figure 11:**
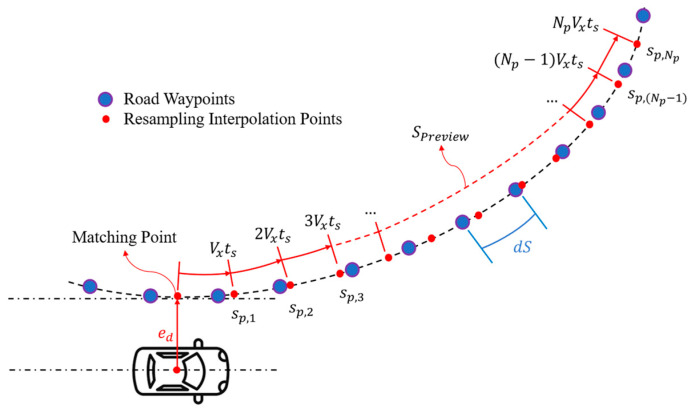
Preview path-station update scheme for NMPC reference generation. The black and red dashed curves denote the reference path and preview sequence, while the blue and red markers represent the road waypoints and resampled points, respectively.

The preview-point update mechanism is illustrated in Figure 11. For the terminal preview path station, an additional extrapolation term is introduced to estimate the distance traveled during the final prediction step:

(86) $${\hat{s}}_{N_{p}}\approx s_{N_{p}}^{*}+t_{s}V_{N_{p}}^{*},$$

where $V_{N_{p}}^{*}$ denotes the predicted vehicle speed at the end of the previous NMPC horizon.

This approximation is valid when the vehicle remains close to the reference path, such that $e_{d}$ and $e_{\psi }$ are small and $\dot{s}\approx V$.

The resulting preview path stations are then used to interpolate the TRO-generated reference velocity, curvature, and other reference quantities along the racetrack.

### 7.4. Reference Output

The controlled output vector contains the vehicle speed, sideslip angle, lateral deviation, and heading-angle error: **y** = ${\left[V,\beta ,e_{d},e_{\psi }\;\right]}^{T}$. At each prediction stage, the reference output is obtained from the TRO trajectory at the previewed path station:

(87) $$y_{k,ref}={\left[\begin{array}{cccc} V_{TRO}\left({\hat{s}}_{k}\right) & \beta_{TRO}\left({\hat{s}}_{k}\right) & 0 & 0 \end{array}\right]}^{T}$$

In this formulation, the vehicle is required to follow the TRO-generated velocity and sideslip-angle profiles, while the lateral deviation and heading-angle error are regulated to zero. Compared with the previous kinematic approximation, using $\beta_{TRO}$ provides a dynamically consistent sideslip-angle reference because it is generated by the offline double-track trajectory optimization model.

### 7.5. NMPC Cost Function

In practical closed-loop operation, discrepancies between the prediction model and the simulated plant, as well as unmodeled disturbances, may cause temporary violations of the imposed constraints. Such violations can make the NMPC optimization problem difficult to solve or even infeasible. To enhance numerical robustness, a nonnegative slack variable $s_{h}$ is introduced to relax the inequality constraints:

(88) $$h_{min}-s_{h,k}\leq h\leq h_{max}+s_{h,k},\;s_{h,k}\geq 0$$

where h contains the tire–road adhesion ellipse constraints and $s_{h,k}$ is the nonnegative slack vector at prediction stage k. The slack variables are introduced to soften the tire–road adhesion ellipse constraints and improve numerical feasibility.

The NMPC cost function is formulated to balance trajectory-tracking accuracy, control smoothness, and constraint relaxation. It consists of the output tracking error, control effort, and slack-variable penalty over the prediction horizon:

(89) $$J_{NMPC}=\sum_{k=1}^{N_{p}}{\frac{1}{2}\left\|\begin{array}{c} S_{y}^{-1}\left(y_{k}-y_{k,ref}\right) \end{array}\right\|}_{Q}^{2}+\sum_{k=0}^{N_{p}-1}{\frac{1}{2}\left\|\begin{array}{c} S_{u}^{-1}u_{k} \end{array}\right\|}_{R}^{2}+\sum_{k=0}^{N_{p}}\frac{1}{2}s_{h,k}^{T}Zs_{h,k}$$

where $S_{y}$ and $S_{u}$ are the scaling matrices for the output variables and control inputs, respectively. The weighting matrices ***Q***, ***R***, and ***Z*** penalize the tracking errors, control efforts, and tire–road adhesion-constraint violations, respectively. The matrix ***Q*** determines the relative importance of velocity tracking, sideslip-angle regulation, lateral deviation, and heading-angle error, whereas the matrix ***R*** is used to suppress aggressive variations in the rear-wheel torque rates and steering-rate input.

To clarify the selection of the NMPC parameters, the sampling time, prediction horizon, weighting matrices, and slack-variable penalties were selected according to both tracking-performance and numerical-feasibility considerations. The sampling time was set to 0.05 s to provide sufficiently fast control updates for high-speed maneuvers, while the prediction horizon was set to 30 steps, corresponding to a preview time of 1.5 s. This preview length provides a compromise between anticipating upcoming curvature changes and maintaining real-time computational feasibility. The output weighting matrix was selected to penalize velocity-tracking error, sideslip angle, lateral deviation, and heading-angle error. The input weighting matrix was used to penalize rear-wheel torque-rate and steering-rate commands, thereby reducing aggressive actuator variations and improving command smoothness. The slack-variable penalty was selected to strongly discourage tire–road adhesion-constraint violations while maintaining numerical feasibility during transient near-limit conditions. A formal Lyapunov-based asymptotic stability proof and recursive feasibility guarantee are not claimed in the present work. Instead, the proposed NMPC is designed to promote practical closed-loop stability and feasibility under the tested high-speed simulation conditions. The cost function penalizes velocity-tracking error, sideslip angle, lateral deviation, and heading-angle error, while the constraints limit actuator commands, motor power, track-boundary violation, and tire–road adhesion utilization. Feasibility is further supported by the TRO-generated feasible reference trajectory, receding-horizon preview, slack variables, warm-starting from the previous solution, and solver-status monitoring. The closed-loop simulation results show bounded tracking errors, no track-boundary violation, and successful solver status for the stable cases. A rigorous terminal-set-based NMPC formulation will be considered in future work.

Table 2 summarizes the resulting NMPC formulation. The optimization problem is solved using acados [31], a high-performance open-source solver for embedded optimal control. In the proposed implementation, the vehicle prediction model is integrated using an implicit Runge–Kutta (IRK) scheme, and the optimal control problem is transcribed using a multiple-shooting strategy. To reduce the computational effort required at each control update, the real-time iteration strategy is adopted. With this strategy, only a single SQP step is carried out during each NMPC sampling period. The NMPC problem definition, integration scheme, transcription settings, weighting matrices, model parameters, and QP solver options are configured through the MATLAB high-level interface. The resulting solver code is automatically generated in C and connected to Simulink through an S-function for closed-loop simulation. The main acados solver settings are provided in Table A3.

## 8. Numerical Results

### 8.1. Simulation Setup

The simulations were conducted on a custom-built desktop computer equipped with an Intel^®^ Core^TM^ i9-12900K processor (Intel Corporation, Santa Clara, CA, USA) and 64 GB RAM. The online control modules shown in Figure 9 were implemented and executed in MATLAB/Simulink R2024b. The proposed framework was evaluated on one lap of the Shanghai International Circuit. The raw track data were obtained from the racetrack database (https://github.com/TUMFTM/racetrack-database, accessed on 5 December 2025), and the time-optimal reference trajectories were generated by the proposed TRO algorithm. The total length of the optimized racing line was 5445 m. This trajectory was discretized with a uniform spatial interval of ds = 1 m for both TRO planning and NMPC tracking. To improve numerical robustness at low speeds, where the vehicle dynamics and their gradients can become sensitive to small longitudinal velocities, the initial vehicle speed in the TRO, NMPC, and Simulink simulations was set to 1 m/s. To evaluate the influence of the prediction horizon on NMPC tracking performance and computational efficiency, $N_{p}$ was varied while the sampling time was fixed at $t_{s}=0.05$ s. Therefore, the total prediction horizon is $T_{p}=N_{p}t_{s}$. Increasing $N_{p}$ increases the preview time and prediction distance, but it also increases the size and nonlinearity of the online optimization problem. For $N_{p}=20$, the prediction horizon is $T_{p}=1.0$ s. At high vehicle speed, this horizon corresponds to a relatively short look-ahead distance. Therefore, the NMPC cannot anticipate the upcoming curvature early enough, and the steering and torque commands are generated too late when the vehicle approaches the corner. As a result, the closed-loop tracking fails. For $N_{p}=30$, the prediction horizon increases to $T_{p}=1.5$ s, which provides sufficient preview information for the controller to prepare the steering and torque commands before entering the corner. This case achieves successful tracking with an RMS lateral error of 0.086 m, a maximum lateral error of 0.282 m, and a maximum solution time of 4.215 ms. For $N_{p}=40$, the prediction horizon becomes $T_{p}=2.0$ s. Although this longer horizon provides more preview information, it also increases the number of decision variables and constraints. Under the RTI scheme, only one SQP iteration is performed at each sampling instant. Therefore, the larger and more nonlinear optimization problem may result in a less fully converged local solution within one RTI step, especially during near-limit cornering. In addition, the longer horizon may make the controller more conservative with respect to future tire-force, actuator, and track-boundary constraints. Consequently, the maximum solution time increases to 9.593 ms, and the tracking error becomes larger, with an RMS lateral error of 0.119 m and a maximum lateral error of 0.503 m. This indicates that the longer horizon does not provide additional benefit for this case. This explanation should be interpreted as an engineering interpretation based on the observed closed-loop behavior and the RTI solver characteristics, rather than as a theoretically proven conclusion regarding prediction-horizon selection. Therefore, $N_{p}=30$ corresponding to $T_{p}=1.5$ s, was selected as the best compromise between prediction distance, tracking accuracy, and real-time computational feasibility. The results are summarized in Table 3.

### 8.2. Trajectory Optimization Results

**Figure 12:**
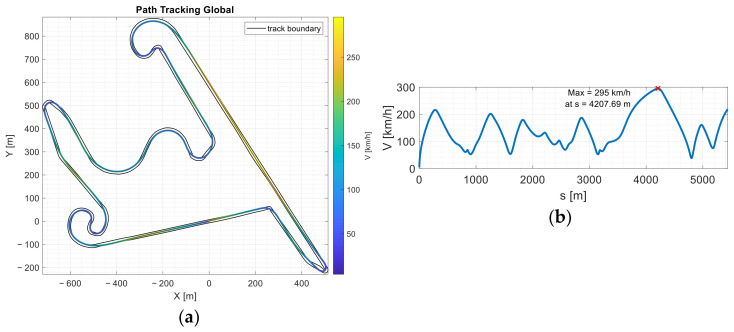
Representative TRO optimization result for $K_{r}$ = 0.5. (**a**) Optimized global trajectory with velocity indicated by the color bar and track boundaries shown in black; (**b**) Optimized velocity profile along the curvilinear coordinate s.

Figure 12 presents the representative TRO result for $K_{r}$ = 0.5, which achieves the shortest lap time among all stable torque distribution cases. The racing line represents the vehicle center of gravity (CG) path, and the color contour indicates the velocity magnitude along the lap.

The optimized trajectories exhibit highly dynamic racing behavior: the vehicle efficiently decelerates before corner apexes, follows a tight and smooth racing line through the turns, and accelerates strongly on corner exits while maintaining high speeds on straight sections. The performance comparison is summarized in Table 4.

The quantitative effects of the virtual 4WD strategy on optimal performance are summarized in Table 4. As the distribution gain $K_{r}$ increases, both lap time and maximum speed improve consistently. At $K_{r}$ = 0.1, the lap time is reduced by 3.63% compared to the pure RWD case ($K_{r}$ = 0). This improvement continues progressively, reaching 5.77% at $K_{r}$ = 0.2, 7.51% at $K_{r}$ = 0.3, and 8.93% at $K_{r}$ = 0.4. The best performance is achieved at $K_{r}$ = 0.5, where the shortest lap time is 163.30 s (a 10.31% reduction) and the maximum speed reaches 295 km/h.

These results show that the proposed rear-dominant virtual 4WD strategy improves lap-time performance by providing additional front-wheel torque assistance and better front–rear tire-force utilization. Since increasing $K_{r}$ also increases the available front-wheel propulsion/braking capability, the improvement should be interpreted as the combined effect of torque distribution and front-motor assistance rather than a purely torque-normalized comparison.

### 8.3. Nonlinear Model Predictive Control Results

#### 8.3.1. Overall Path-Tracking Performance

**Figure 13:**
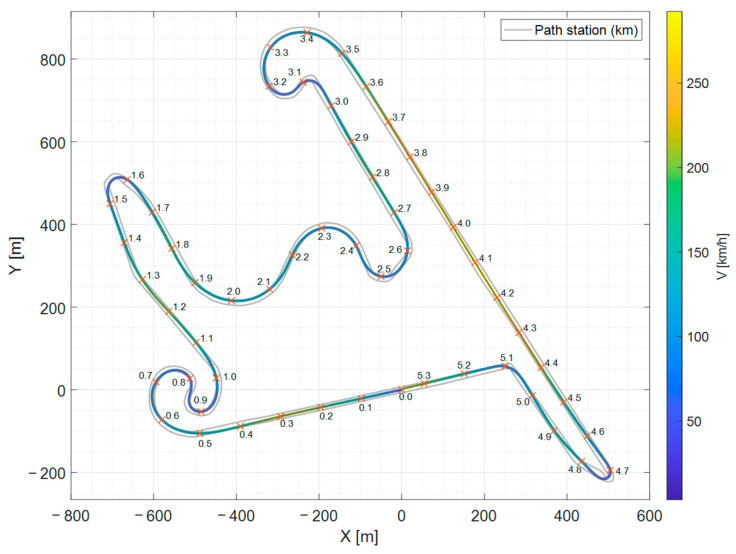
Representative NMPC path-tracking result for $K_{r}$ = 0.5.

Figure 13 presents the representative NMPC path-tracking result for $K_{r}$ = 0.5, which provides the best compromise between lap-time performance and tracking accuracy among all stable cases. The vehicle successfully follows the TRO-generated racing trajectory while remaining within the track boundaries. The velocity contour indicates that the vehicle decelerates before corner apexes, accelerates during corner exits, and maintains high speeds on straight sections.

**Figure 14:**
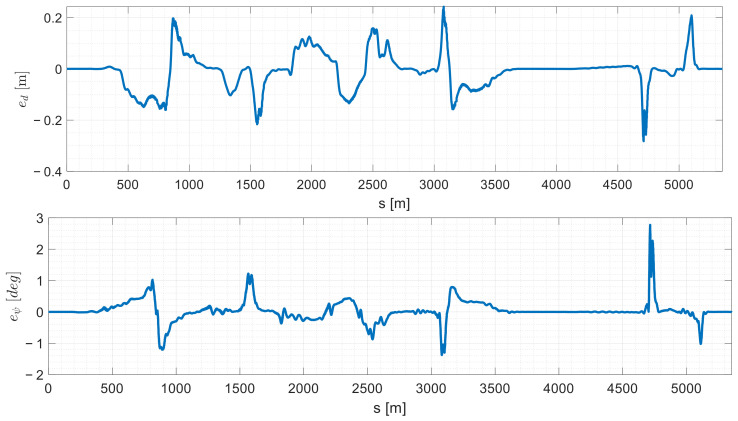
Representative path-tracking errors for $K_{r}$ = 0.5.

Figure 14 presents the tracking-error responses for the representative $K_{r}$ = 0.5 case. The proposed NMPC controller uses predicted vehicle states and previewed reference trajectories to coordinate the longitudinal and lateral vehicle motions, thereby maintaining small tracking errors along the entire racetrack. The RMS lateral deviation is 0.086 m, with a maximum value of 0.282 m. Meanwhile, the RMS and maximum heading-angle errors are 0.466 deg and 2.776 deg, respectively.

**Figure 15:**
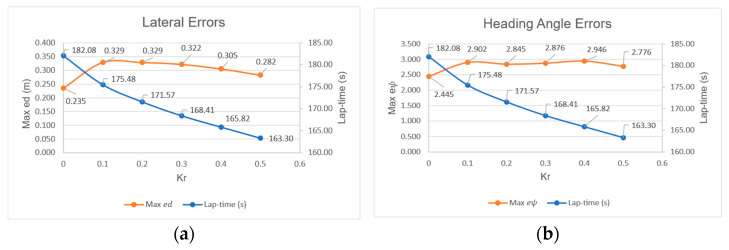
Comparison of maximum tracking errors and lap time under different torque distribution gains. (**a**) Maximum lateral deviation and corresponding lap time; (**b**) Maximum heading-angle error and corresponding lap time.

Figure 15 further illustrates the trade-off between lap-time performance and path-tracking accuracy under different torque distribution gains. As $K_{r}$ increases from 0 to 0.5, the lap time decreases continuously due to the enhanced longitudinal traction provided by the front wheels. However, the tracking errors do not follow a monotonic trend. For intermediate values of $K_{r}$, the front tires are required to generate both longitudinal driving force and lateral steering force. According to the tire–road adhesion ellipse constraint, the increased longitudinal force reduces the available lateral force, which can degrade path-tracking accuracy.

Although the pure rear-wheel-drive case ($K_{r}$ = 0) achieves the smallest tracking errors, it also results in the longest lap time. In contrast, $K_{r}$ = 0.5 achieves the shortest lap time while reducing the maximum lateral and heading-angle errors compared with the intermediate cases ($K_{r}$ = 0.1~0.4). This indicates that a moderate level of front-wheel torque assistance improves the balance of front–rear tire force utilization. At $K_{r}$ = 0.5, the front-wheel traction is sufficient to reduce rear tire saturation and improve yaw stability, resulting in the best compromise between lap-time reduction and tracking performance among the stable cases.

Table 5 compares the TRO and NMPC performance for different virtual 4WD torque distribution gains, $K_{r}$. As $K_{r}$ increases from 0 to 0.5, both TRO and NMPC lap times decrease, indicating improved longitudinal traction and lap-time performance. The NMPC lap time is reduced from 183.13 s to 163.73 s, while the maximum lateral deviation remains below 0.33 m and the maximum heading-angle error stays below 3 deg. Therefore, $K_{r}$ = 0.5 provides the best trade-off between lap-time reduction and tracking accuracy among the stable cases.

To evaluate whether the tire-model mismatch affects the trackability of the TRO-generated reference, the closed-loop tracking performance was examined. For the representative case with $K_{r}=0.5$, the RMS lateral error is 0.086 m, the maximum lateral error is 0.282 m, and the lap-time difference between TRO and NMPC is 0.43 s. In addition, the tire working-point results show that the tires operate close to the tire–road adhesion boundary during near-limit cornering. These results indicate that, although a reduced tire model is used in the online NMPC, the TRO-generated reference remains trackable under the considered simulation conditions.

For the representative stable case $K_{r}=0.5$, the maximum lateral error occurs at s = 4711 m. At this location, the vehicle deviates toward the right boundary, and the available right-side margin after subtracting the reserved clearance is 4.145 m. Therefore, the maximum lateral error of 0.282 m corresponds to 6.81% of the available margin. This confirms that the vehicle remains inside the track-boundary constraint for the $K_{r}=0.5$ case. The corresponding values for the other $K_{r}$ cases are summarized in Table 6.

Table 7 shows the performance of TRO and NMPC when the torque distribution gain is increased to $K_{r}=0.6$. At this value, although the offline TRO can still generate a feasible trajectory, the online NMPC fails to track it, with large spikes observed in both lateral deviation and heading error. The lap time from TRO is further increased compared to lower gains. This tracking failure is caused by the saturation of the front tire–road adhesion capacity due to excessive longitudinal force, which significantly reduces the available lateral force and leads to strong understeer behavior. These results confirm that $K_{r}=0.5$ represents the practical upper limit for stable and effective operation of the proposed virtual 4WD NMPC framework.

**Figure 16:**
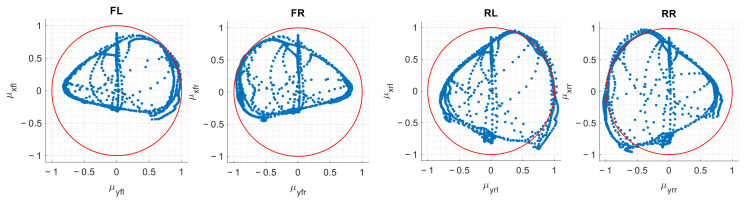
Normalized tire working points for the $K_{r}$ = 0.5 case. The blue markers represent the normalized tire operating points during the simulation, while the red circle denotes the normalized tire–road adhesion limit.

**Figure 17:**
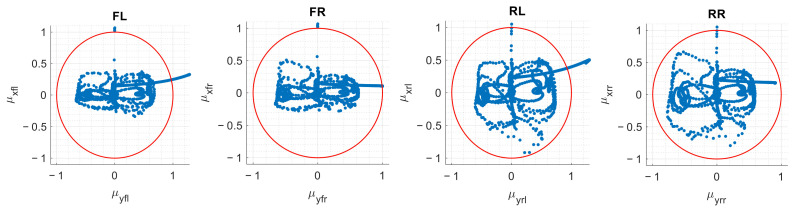
Normalized tire working points for the $K_{r}$ = 0.6 case. The blue markers represent the normalized tire operating points during the simulation, while the red circle denotes the normalized tire–road adhesion limit.

When $K_{r}$ increases from 0.5 to 0.6, the proportional front-wheel driving torque becomes larger. This increases the normalized front longitudinal tire-force demand. Under the tire–road adhesion ellipse constraint, a higher longitudinal force demand reduces the remaining lateral-force reserve of the front tires. Therefore, the front axle cannot generate sufficient lateral force during high-curvature sections. This produces understeer behavior, increases the lateral tracking error, and eventually leads to path-tracking failure. This mechanism is illustrated by the tire working-point comparison in Figure 16 and Figure 17. For the $K_{r}$ = 0.5 case, Figure 16 shows that the tire operating points remain close to the adhesion boundary while maintaining sufficient lateral-force reserve for stable tracking.

In contrast, for the $K_{r}$ = 0.6 case, Figure 17 shows more unfavorable operating points with high tire-force utilization and reduced front-axle lateral-force reserve. These results indicate that the increased front-wheel driving demand consumes more available tire-force capacity at the front axle, resulting in degraded cornering capability and path-tracking failure.

#### 8.3.2. Vehicle States

**Figure 18:**
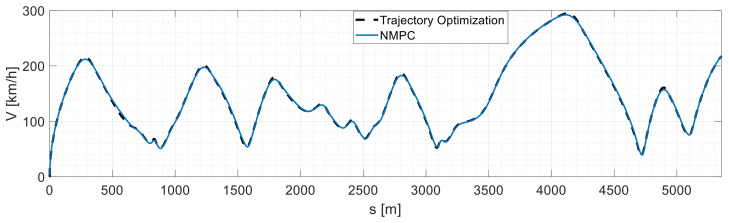
Velocity-tracking response for the representative $K_{r}$ = 0.5 case. The black dashed line represents the TRO-optimized velocity profile, while the blue solid line denotes the NMPC tracking response.

Figure 18 compares the TRO-optimized velocity profile and the corresponding NMPC tracking response for the representative $K_{r}$ = 0.5 case. The NMPC controller closely follows the near-limit velocity profile generated by the TRO layer throughout the lap. The NMPC lap time is 163.73 s, which is only 0.43 s longer than the TRO optimum of 163.30 s, corresponding to a relative error of 0.26%.

For the remaining stable cases, the NMPC lap times are 183.13 s, 176.50 s, 171.79 s, 168.61 s, and 166.27 s for $K_{r}$ = 0, 0.1, 0.2, 0.3, and 0.4, respectively. The corresponding relative differences with respect to the TRO solutions are 0.58%, 0.59%, 0.13%, 0.12%, and 0.27%. These small differences indicate that the proposed NMPC controller can effectively track the aggressive TRO-generated trajectories while maintaining stable vehicle behavior under the imposed tire and track constraints.

**Figure 19:**
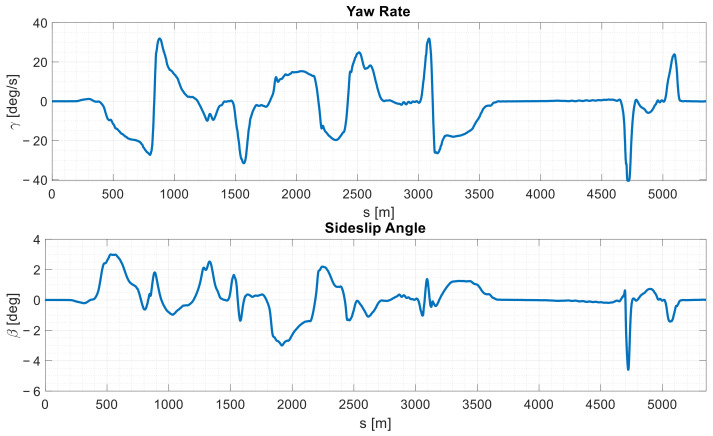
Vehicle yaw-rate and sideslip-angle responses for the representative $K_{r}$ = 0.5 case.

Figure 19 illustrates the yaw-rate and sideslip-angle responses for $K_{r}$ = 0.5. The NMPC controller successfully regulated the vehicle yaw motion to follow the aggressive path curvatures generated by the TRO layer while maintaining the vehicle sideslip angle within a stable operating range. This indicates that the NMPC controller maintains lateral stability while tracking a near-limit trajectory.

#### 8.3.3. Torque and Steering Commands

**Figure 20:**
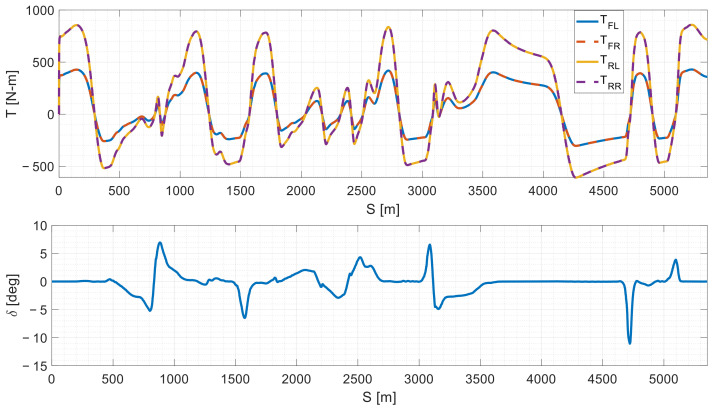
Control input responses for the representative $K_{r}$ = 0.5 case. The upper plot shows the optimized rear-wheel torque commands and the generated front-wheel torques obtained from the virtual 4WD distribution law. The lower plot shows the front-wheel steering command.

Figure 20 presents the wheel-torque and steering-command responses generated by the proposed NMPC controller and the virtual 4WD distribution law. The rear-wheel torque commands and steering input remain smooth throughout the lap without significant oscillations, indicating that the proposed controller produces actuator-friendly commands. Since the NMPC optimizes the rates of the rear-wheel torque commands and steering input, the corresponding rear-wheel torque and steering commands remain smooth. The front-wheel torques are then generated separately using the proposed gain-based virtual 4WD distribution law.

#### 8.3.4. Execution Performance and Computational Complexity

To verify the real-time feasibility of the RTI-based NMPC implementation, solver statistics were recorded over the complete lap for the representative stable case $K_{r}$ = 0.5. Since the RTI scheme performs only one SQP iteration at each sampling instant, the solver behavior was evaluated using solver status, SQP/RTI iteration number, computation time, and KKT residuals. The results are summarized in Table 8. For this representative stable case, the solver maintained a high success rate and the computation time remained below the NMPC sampling time of 0.05 s. This indicates that the proposed reduced-complexity NMPC formulation is suitable for real-time-oriented closed-loop simulation under the considered high-speed racing conditions.

The NMPC solver achieved a 100% success rate with zero solver failures over the complete lap. The mean and maximum computation times were 1.912 ms and 4.215 ms, respectively, which are significantly lower than the sampling period of 50 ms. The SQP/RTI iteration number remained equal to one, as expected from the real-time iteration scheme. Because the RTI scheme performs only one SQP step at each sampling instant, the reported KKT residuals should be interpreted as diagnostic indicators of RTI solution quality rather than full-convergence metrics. The equality residual remains small, indicating that the discretized system dynamics are well satisfied. The larger peak stationarity, inequality, and complementarity residuals occur during aggressive near-limit cornering, where tire-force and actuator constraints are highly active. Therefore, the acceptability of the RTI solution is assessed together with the 100% solver success rate, zero solver failures, bounded tracking errors, track-boundary satisfaction, and tire working-point analysis.

To further quantify the computational benefit of the reduced-dimensional NMPC formulation, Table 9 compares the proposed NMPC with a conventional full four-wheel torque NMPC structure. The conventional full four-wheel torque NMPC used in this comparison is an internally implemented baseline constructed within the same modeling, discretization, and RTI-based acados framework. It is not intended to reproduce a specific controller from the literature. Instead, it provides a controlled baseline for evaluating the effect of replacing four independent wheel-torque decision variables with the proposed rear-dominant virtual 4WD torque distribution structure. Both formulations were evaluated using the same sampling time, prediction horizon, racing scenario, and RTI-based acados implementation. In the proposed formulation, the online NMPC optimizes the steering-rate input and the rear-left/rear-right torque-rate inputs, resulting in $n_{x}$ = 11 and $n_{u}$ = 3. For $N_{p}$ = 30, the number of main state/input decision variables is $\left(N_{p}+1\right)n_{x}+N_{p}n_{u}=431$. In contrast, the conventional full four-wheel torque NMPC treats the front-left, front-right, rear-left, and rear-right torque commands as independent online decision variables, leading to $n_{x}$ = 13, $n_{u}$ = 5, and 553 main state/input decision variables.

As shown in Table 9, the proposed formulation reduces the main state/input decision-variable count from 553 to 431, corresponding to a 22.1% reduction. The number of dynamic equality constraints is reduced from 390 to 330, while the number of input-rate decision variables decreases from 150 to 90. The execution-time comparison shows that the mean NMPC computation time decreases from 4.336 ms to 1.912 ms, and the maximum computation time decreases from 7.377 ms to 4.215 ms. These correspond to reductions of approximately 55.9% and 42.9%, respectively. Both formulations achieved a 100% solver success rate with zero solver failures in the evaluated simulation. These results indicate that the proposed rear-dominant virtual 4WD strategy reduces the online optimization dimension and improves solver execution time under the investigated simulation conditions. Since the memory requirement was not separately measured, quantitative memory savings are not claimed and will be investigated in future work.

It should be noted that the reduced computational burden is obtained at the cost of reducing the feasible input space. In a fully independent four-wheel torque NMPC, the front-left, front-right, rear-left, and rear-right wheel torques can be selected independently. In contrast, the proposed rear-dominant virtual 4WD strategy constrains the front-wheel torques through $T_{fl}=K_{r}T_{rl}$ and $T_{fr}=K_{r}T_{rr}$. Therefore, the feasible torque-allocation space of the proposed formulation is a subset of that of a fully independent four-wheel torque allocation strategy. This trade-off improves computational efficiency and real-time feasibility, but it may lead to suboptimal torque allocation under rapidly varying tire-load or adhesion conditions.

## 9. Conclusions

This paper proposed a rear-dominant virtual 4WD trajectory optimization and NMPC framework for high-speed autonomous vehicle path tracking near the handling limits. The offline TRO layer generates a minimum-lap-time racing trajectory using a double-track vehicle model with tire-force and track-boundary constraints. The online NMPC controller then tracks this trajectory using the steering command and the rear-left/rear-right torque commands.

To exploit the traction capability of the four-wheel-drive vehicle while maintaining a reduced-complexity control structure, a virtual 4WD torque distribution strategy was introduced. The proposed NMPC controller optimized only the rear-left and rear-right wheel torque commands and the steering input in real time, while the front-wheel torques were generated through a proportional gain-based distribution law. By incorporating previewed reference trajectories and predicted vehicle motion, the NMPC controller successfully tracked the aggressive TRO-generated racing trajectories while maintaining stable vehicle behavior under highly dynamic driving conditions.

Simulation results on the Shanghai International Circuit demonstrated that increasing the torque distribution gain improved the vehicle longitudinal traction capability and reduced the lap time. As $K_{r}$ increased from 0 to 0.5, the TRO lap time decreased from 182.08 s to 163.30 s, corresponding to a 10.31% reduction. The NMPC controller successfully tracked the TRO-generated near-limit trajectory, and the representative $K_{r}$ = 0.5 case achieved a lap time of 163.73 s, which was only 0.43 s longer than the TRO optimum. Among all stable cases, $K_{r}$ = 0.5 achieved the best compromise between lap-time performance and tracking accuracy, indicating a more balanced front–rear tire force utilization and improved overall vehicle stability. However, excessive front-wheel longitudinal force reduced the available lateral tire force because of the tire–road adhesion ellipse limitation, leading to degraded path-tracking performance and eventual path-following failure for large $K_{r}$ values.

The proposed method still has some limitations. The present study is a simulation-based investigation, and the proposed TRO–NMPC framework was evaluated through closed-loop numerical simulations on the Shanghai International Circuit. No experimental vehicle tests using a real vehicle platform were conducted in this work. In addition, the rear-dominant virtual 4WD strategy uses a fixed proportional front-torque distribution gain, which reduces the online optimization dimension but does not provide fully independent four-wheel torque allocation and may become suboptimal under rapidly varying tire-load and adhesion conditions. Moreover, the steering system is represented by the front-wheel steering angle with steering-angle bounds, steering-rate bounds, and steering-rate penalties rather than by an explicit steering actuator model that includes stiffness, damping, and actuator dynamics. Although the NMPC formulation promotes practical closed-loop feasibility through state/input constraints, tire–road adhesion constraints, slack variables, warm-starting, and solver-status monitoring, a formal Lyapunov-based stability proof with terminal constraints is beyond the scope of this work. Therefore, the reported results demonstrate the feasibility and performance of the proposed control framework in simulation, while future work will focus on experimental validation, adaptive or optimized torque-distribution strategies, more detailed actuator modeling, and the development of stability-guaranteed NMPC formulations for high-speed autonomous racing.

Overall, under the investigated simulation conditions on the Shanghai International Circuit, the proposed rear-dominant virtual 4WD TRO–NMPC framework reduced the simulated lap time while maintaining bounded tracking errors, satisfying the track-boundary constraints, and achieving successful solver status. These results demonstrate the feasibility of the proposed framework in closed-loop numerical simulation. Future work will focus on hardware-in-the-loop testing, real-vehicle validation, adaptive or optimized torque-distribution strategies, more detailed actuator modeling, and stability-guaranteed NMPC formulations.

## Figures and Tables

**Table 1:** Decision variables, constraints, and bounds used in the TRO problem.

	Description	Symbol/ Equation	Scale	Lower	Upper	Units
**Objective**	**Objective function**	$J_{TRO}$ (76)				
**Optimization variables**						
State variables	Velocity	V	100	1	360/3.6	m/s
	Sideslip angle	β	1	−π/4	π/4	rad
	Yaw rate	γ	1	−π/2	π/2	rad/s
	Wheel velocities	$\omega_{i}$	100/R	0	360/3.6/R	rad/s
	Lateral deviation	$e_{d}$	5	$-{Ɲ}_{r}+\left(\frac{w_{t}}{2}+w_{s}\right)$	${Ɲ}_{l}-\left(\frac{w_{t}}{2}+w_{s}\right)$	m
	Relative heading	$e_{\psi }$	1	−π/4	π/4	rad
Control variables	Left rear wheel torque	$T_{rl}$	2000	−2000	2000	N·m
	Right rear wheel torque	$T_{rr}$	2000	−2000	2000	N·m
	Front-wheel steering angle	δ	π/8	−π/8	π/8	rad
Auxiliary variables	Longitudinal acceleration	${\bar{a}}_{x}$	g	−3g	3g	$m/s^{2}$
	Lateral acceleration	${\bar{a}}_{y}$	g	−3g	3g	$m/s^{2}$
Track parameter	Path curvature	C	−	−	−	1/m
**Subject to**						
Constraints	Collocation dynamics	(69),(70)	−	−	−	−
	Acceleration consistency	(22),(23)	g	$-10^{-3}$	$10^{-3}$	−
	Tire–road adhesion ellipse	(51)	1	0	1	−
	Motor-power constraint	(52)	150	−150	150	kW
Rate bounds	Rate of left rear wheel torque	${\dot{T}}_{rl}$	5000	−5000	5000	N·m/s
	Rate of right rear wheel torque	${\dot{T}}_{rr}$	5000	−5000	5000	N·m/s
	Rate of wheel steering angle	$\dot{\delta }$	π/8	−π/8	π/8	rad/s

**Table 2:** Decision variables, bounds, and constraints used in the NMPC problem.

	Description	Symbol/ Equation	Scale	Lower	Upper	Units
**Objective**	**Objective function**	$J_{NMPC}$ (89)				
**Optimization variables**						
**State variables**	Velocity	V	100	1	360/3.6	m/s
	Sideslip angle	β	1	−π/4	π/4	rad
	Yaw rate	γ	1	−π/2	π/2	rad/s
	Longitudinal acceleration	${\bar{a}}_{x}$	g	−3g	3g	$m/s^{2}$
	Lateral acceleration	${\bar{a}}_{y}$	g	−3g	3g	$m/s^{2}$
	Path station	*s*	1	0	Inf	m
	Lateral deviation	$e_{d}$	5	$-{Ɲ}_{r}+\left(\frac{w_{t}}{2}+w_{s}\right)$	${Ɲ}_{l}-\left(\frac{w_{t}}{2}+w_{s}\right)$	m
	Relative heading	$e_{\psi }$	1	−π/4	π/4	rad
	Left rear wheel torque	$T_{rl}$	2000	−2000	2000	N·m
	Right rear wheel torque	$T_{rr}$	2000	−2000	2000	N·m
	Front-wheel steering angle	δ	π/8	−π/8	π/8	rad
	Slack variable	$s_{h}$	1	0	Inf	−
**Control inputs**	Rate of left rear wheel torque	${\dot{T}}_{rl}$	5000	−5000	5000	N·m/s
	Rate of right rear wheel torque	${\dot{T}}_{rr}$	5000	−5000	5000	N·m/s
	Rate of wheel steering angle	$\dot{\delta }$	π/8	−π/8	π/8	rad/s
**Preview** **parameter**	Path curvature	C	−	−	−	1/m
**Constraints**	IRK-discretized dynamics	(84)	−	−	−	−
	Initial-state constraint	(83)	−	−	−	−
	Tire–road adhesion ellipse	(51)	1	0	1	−
	Motor-power constraint	(53)–(56)	150	−150	150	kW

**Table 3:** Sensitivity analysis of NMPC prediction horizon length for the $K_{r}$ = 0.5 case.

$K_{r}$	$N_{p}$	$T_{p}$	$t_{s}$	Max $t_{sol\;}$(ms)	RMS $e_{d}$ (m)	Max $\left|e_{d}\right|$ (m)	Closed-Loop Status
0.5	20	1.0	0.05	−	−	−	Tracking failed
0.5	30	1.5	0.05	4.215	0.086	0.282	Successful
0.5	40	2.0	0.05	9.593	0.119	0.503	Successful

**Table 4:** Performance comparison of optimized trajectories for different torque distribution gains.

Distribution Gain	Lap-Time (s)	Maximum Speed (km/h)	Lap-Time Reduction (%)
$K_{r}$ = 0	182.08	249	0.00
$K_{r}$ = 0.1	175.48	262	3.63
$K_{r}$ = 0.2	171.57	272	5.77
$K_{r}$ = 0.3	168.41	280	7.51
$K_{r}$ = 0.4	165.82	288	8.93
$K_{r}$ = 0.5	163.30	295	10.31

**Table 5:** TRO and NMPC performance for different $K_{r}$ values.

$K_{r}$	TRO Lap-Time (s)	NMPC Lap-Time (s)	Max$\left|e_{d}\right|$ (m)	RMS $e_{d}$ (m)	Max$\left|e_{\psi }\right|$ (deg)	RMS $e_{\psi }$ (deg)
0	182.08	183.13	0.235	0.063	2.445	0.442
0.1	175.48	176.50	0.329	0.085	2.902	0.455
0.2	171.57	171.79	0.329	0.084	2.845	0.459
0.3	168.41	168.61	0.322	0.087	2.876	0.461
0.4	165.82	166.27	0.305	0.084	2.946	0.467
0.5	163.30	163.73	0.282	0.086	2.776	0.466

**Table 6:** Relationship between maximum lateral error and available track margin for different $K_{r}$ values.

$K_{r}$	Maximum Lateral Error (m)	Available Margin at Maximum-Error Point (m)	Error/Margin Ratio (%)	Boundary Satisfied
0	0.24	8.17	2.94	Yes
0.1	0.33	4.48	7.37	Yes
0.2	0.33	4.84	6.82	Yes
0.3	0.32	3.99	8.02	Yes
0.4	0.31	4.64	6.68	Yes
0.5	0.282	4.145	6.81	Yes

**Table 7:** Failure case analysis for excessive torque distribution gain.

$K_{r}$	TRO Result	NMPC Result	Reason
0.6	197.02 s	Failed	Front tire–road adhesion capacity saturation leading to strong understeer

**Table 8:** NMPC RTI solver statistics for $K_{r}$ = 0.5.

Quantity	Mean	Max	Unit
NMPC computation time $t_{sol}$	1.912	4.215	ms
SQP/RTI iteration	1.00	1.00	−
KKT stationarity residual	1.309 × 10^−2^	2.296 × 10^1^	−
KKT equality residual	1.018 × 10^−4^	4.357 × 10^−3^	−
KKT inequality residual	2.773 × 10^−3^	1.074	−
KKT complementarity residual	2.694 × 10^−3^	8.535	−
Solver success rate	100.00	−	%
Solver failure count	0	−	−

**Table 9:** Structural complexity and execution-performance comparison between the proposed reduced-dimensional NMPC and a conventional full four-wheel torque NMPC.

Quantity	Proposed Reduced- Dimensional NMPC	Conventional Full Four-Wheel Torque NMPC	Difference	Unit
Optimized torque commands	2 rear-wheel torque commands	4 independent wheel-torque commands	Reduced	−
Generated torque commands	Front-left and front-right by virtual distribution law	None	−	−
State dimension, ($n_{x}$)	11	13	−2	−
Input dimension, ($n_{u}$)	3	5	−2	−
Prediction horizon, ($N_{p}$)	30	30	Same	−
Main state/input decision variables	431	553	22.1% reduction	−
Dynamic equality constraints	330	390	15.4% reduction	−
Input-rate decision variables	90	150	40.0% reduction	−
Mean NMPC computation time, ($t_{sol}$)	1.912	4.336	55.9% reduction	ms
Maximum NMPC computation time, ($t_{sol}$)	4.215	7.377	42.9% reduction	ms
SQP/RTI iteration	1.00	1.00	Same	−
Solver success rate	100.00	100.00	Same	%
Solver failure count	0	0	Same	−

## Data Availability

The racetrack data used in this study are available from the TUMFTM racetrack database. The simulation data generated in this study are available from the corresponding authors upon reasonable request.

## Appendix A. Numerical Parameters for Reproduction

The main vehicle, tire, aerodynamic, trajectory-optimization, NMPC, and solver parameters used in this study are summarized in Appendix A to support independent reproduction of the proposed TRO–NMPC framework. The yaw moment of inertia, center-of-gravity height, effective tire rolling radius, and vehicle frontal area were adopted from the CarSim vehicle parameter set used as the representative vehicle model; no empirical equations were used to calculate these parameters. The effective tire rolling radius corresponds to the 205/45 R17 tire size defined in the CarSim tire/vehicle configuration. In Appendix A, m denotes the vehicle mass, $I_{z}$ the yaw moment of inertia, h the center-of-gravity height, $I_{w}$ the wheel rotational inertia, R the effective tire rolling radius, A the frontal area, ρ the air density, $C_{d}$ the aerodynamic drag coefficient, and $C_{l}$ the aerodynamic lift coefficient. The torque distribution gain $K_{r}$ defines the proportional relationship between the generated front-wheel torques and the optimized rear-wheel torque commands, while the NMPC weighting matrices Q, R, and Z penalize the output-tracking error, control-rate input, and slack-variable violation, respectively.

**Table A1:** Vehicle, aerodynamic, and tire-model parameters used in this study.

	Description	Symbol	Value	Unit
**Env** **.**	Gravitational acceleration	g	9.81	$m{/s}^{2}$
	Road–tire adhesion coeff.	$\mu_{0}$	1.0	−
	Air density	ρ	1.2	$kg/m^{3}$
	Aerodynamic drag coeff.	$C_{d}$	0.3	−
	Aerodynamic lift coeff.	$C_{l}$	−0.6	−
**Vehicle**	Front area	*A*	2.2	$m^{2}$
	Total vehicle mass	*m*	1000	kg
	Yaw moment of inertia	$I_{z}$	1050	kg·$m^{2}$
	Distance from CG to front axle	$l_{f}$	1.015	m
	Distance from CG to rear axle	$l_{r}$	1.895	m
	Center-of-gravity height	*h*	0.54	m
	Vehicle track width	$w_{t}$	1.5	m
	Wheel rotational inertia	$I_{w}$	1.2	kg·$m^{2}$
	Effective rolling radius	*R*	0.3	m
**Tire**	Longitudinal stiffness factor	$B_{x}$	18	−
	Longitudinal shape factor	$C_{x}$	1.3	−
	Longitudinal peak-factor linear coeff.	$d_{1x}$	0.95	−
	Longitudinal peak-factor constant coeff.	$d_{2x}$	320	N
	Lateral stiffness factor	$B_{y}$	13	−
	Lateral shape factor	$C_{y}$	1.5	−
	Lateral peak-factor linear coeff.	$d_{1y}$	0.95	−
	Lateral peak-factor constant coeff.	$d_{2y}$	320	N
	Maximum longitudinal adhesion coeff.	$\mu_{x,max}$	1.0	−
	Maximum lateral adhesion coeff.	$\mu_{y,max}$	1.0	−
	Combined-slip regularization constant	$\varepsilon_{\sigma }$	$10^{-4}$	−

**Table A2:** TRO optimization parameters, bounds, and solver settings.

	Description	Symbol	Value	Unit
**Track**	Optimized racing-line length	L	5445	m
**Discretization**	Spatial grid interval	ds	1	m
	Number of spatial intervals	N	5445	−
	Collocation method	−	Direct orthogonal collocation	−
	Collocation order	q	3	−
**Problem setup**	Initial vehicle speed	$V_{0}$	1	m/s
**Bounds**	Vehicle speed	V	[1, 360/3.6]	m/s
	Sideslip angle	β	[−π/4, π/4]	rad
	Yaw rate	γ	[−π/2, π/2]	rad/s
	Wheel angular velocity	$\omega_{i}$	[0, (360/3.6)/R]	rad/s
	Lateral deviation	$e_{d}$	Track-boundary dependent	m
	Relative heading angle	$e_{\psi }$	[−π/4, π/4]	rad
	Rear-left/right wheel torque	$T_{rl}$, $T_{rr}$	[−2000, 2000]	N·m
	Front-wheel steering angle	δ	[−π/8, π/8]	rad
	Longitudinal acceleration	${\bar{a}}_{x}$	[−3g, 3g]	$m{/s}^{2}$
	Lateral acceleration	${\bar{a}}_{y}$	[−3g, 3g]	$m{/s}^{2}$
**Constraints**	Tire–road adhesion ellipse	−	[0, 1]	−
	Wheel motor power	$P_{i}$	[−150, 150]	kW
**Weighting** **factor**	Weight matrix for control increments	R	diag(1, 1, 10)	−
	Weight matrix for auxiliary-variable increments	W	diag(1, 1)	−
**Rate bounds**	Rear-left/right torque rate	${\dot{T}}_{rl}$, ${\dot{T}}_{rr}$	[−5000, 5000]	N·m/s
	Steering-angle rate	$\dot{\delta }$	[−π/8, π/8]	rad/s
**Solver**	Modeling framework	−	CasADi	−
	NLP solver	−	IPOPT	−
	Maximum iterations	−	1000	−
	Convergence tolerance	−	$10^{-3}$	−
	Acceptable tolerance	−	$10^{-6}$ (IPOPT default)	−

Note: The TRO weighting matrices R and W penalize the spatial increments of the control inputs and auxiliary variables, respectively.

**Table A3:** NMPC parameters, weighting matrices, bounds, and acados solver settings.

	Description	Symbol	Value	Unit
**Prediction setup**	Sampling time	$t_{s}$	0.05	s
	Prediction horizon length	$N_{p}$	30	−
	Total prediction time	$T_{p}=N_{p}t_{s}$	1.5	s
**Bounds**	Vehicle speed	V	[1, 360/3.6]	m/s
	Sideslip angle	β	[−π/4, π/4]	rad
	Yaw rate	γ	[−π/2, π/2]	rad/s
	Longitudinal acceleration	${\bar{a}}_{x}$	[−3g, 3g]	$m{/s}^{2}$
	Lateral acceleration	${\bar{a}}_{y}$	[−3g, 3g]	$m{/s}^{2}$
	Lateral deviation	$e_{d}$	Track-boundary dependent	m
	Relative heading angle	$e_{\psi }$	[−π/4, π/4]	rad
	Rear-left/right wheel torque	$T_{rl}$, $T_{rr}$	[−2000, 2000]	N·m
	Front-wheel steering angle	δ	[−π/8, π/8]	rad
**Rate bounds**	Rear-left/right torque rate	${\dot{T}}_{rl}$, ${\dot{T}}_{rr}$	[−5000, 5000]	N·m/s
	Steering-angle rate	$\dot{\delta }$	[−π/8, π/8]	rad/s
**Constraints**	Tire–road adhesion ellipse	−	[0, 1]	−
	Wheel motor power	$P_{i}$	[−150, 150]	kW
**Scaling**	Output scaling matrix	$S_{y}$	diag(1, 0.05, 0.1, 0.05)	−
	Input scaling matrix	$S_{u}$	diag(5000, 5000, π/8)	−
**Weighting** **factor**	Output-tracking weight	**Q**	diag(1, 1, 1, 1)	−
	Control-rate weight	**R**	diag(1, 1, 10)	−
	Slack-variable penalty	**Z**	diag(10, 10, 10, 10)	−
**acados solver**	NLP solver type	−	SQP_RTI	−
	QP solver	qp_solver	full_condensing_hpipm	
	Integrator type	sim_method	IRK	−
	Number of IRK stages	sim_method_num_stages	2	−
	Number of integration steps	sim_method_num_steps	1	−
	Maximum NLP iterations	nlp_solver_max_iter	30	−
	Stationarity tolerance	nlp_solver_tol_stat	$10^{-4}$	−
	Equality tolerance	nlp_solver_tol_eq	$10^{-4}$	−
	Inequality tolerance	nlp_solver_tol_ineq	$10^{-4}$	−
	Complementarity tolerance	nlp_solver_tol_comp	$10^{-4}$	−
	Exact Hessian option	nlp_solver_exact_hessian	true	−
**Implementation**	Code generation	−	C code generated by acados	−
	Closed-loop interface	−	Simulink S-function	−
